# Elevated SFXN2 limits mitochondrial autophagy and increases iron-mediated energy production to promote multiple myeloma cell proliferation

**DOI:** 10.1038/s41419-022-05272-z

**Published:** 2022-09-26

**Authors:** Ying Chen, Jinjun Qian, Pinggang Ding, Wang Wang, Xinying Li, Xiaozhu Tang, Chao Tang, Ye Yang, Chunyan Gu

**Affiliations:** 1 Nanjing Hospital of Chinese Medicine affiliated to Nanjing University of Chinese Medicine, Nanjing, China; 2 School of Medicine & Holistic Integrative Medicine, Nanjing University of Chinese Medicine, Nanjing, China

**Keywords:** Myeloma, Myeloma

## Abstract

Human sideroflexin 2 (SFXN2) belongs to the SFXN protein family, which is a mitochondrial outer membrane protein involved in mitochondrial iron metabolism. Mitochondria are indispensable for cellular energy production and iron metabolism. However, it remains elusive how SFXN2 modulates mitochondrial homeostasis and cellular iron metabolism in multiple myeloma (MM). In this study, we first found that SFXN2 was significantly elevated and correlated to poor outcomes in MM patients from clinical datasets. SFXN2 overexpression promoted MM cell proliferation and suppressed starvation-induced autophagy/mitophagy, while SFXN2 knockdown aggravated mitochondria damage and autophagic processes in ARP1 and H929 MM cell lines. Furthermore, inhibition of SFXN2 exerted effectively anti-myeloma activity in vivo by using myeloma xenograft model. Mechanism studies indicated that heme oxygenase 1 (HO1) with anti-oxidant function contributed to the process of autophagy suppression and cellular proliferation mediated by SFXN2. Our study revealed the critical role of SFXN2 in regulating mitochondrial bioenergetics, mitophagy, cellular iron metabolism, and redox homeostasis in interconnected and intricate way. Collectively, these findings not only provide insights into the metabolic reprogramming of tumor cells, but also highlight the therapeutic potential of SFXN2 in combination with iron metabolism as target for prognosis and treatment in MM patients.

## Introduction

Multiple myeloma (MM) is a common hematological malignancy worldwide characterized by the clonal expansion of malignant plasma cells, which typically secret monoclonal immunoglobulin (termed as “M” protein) thus sustain high endoplasmic reticulum (ER) stress [1, 2]. Despite considerable improvements in treatments from proteasome inhibitors to immune modulators, MM remains incurable [3]. The pathogenesis and progression mechanisms of MM are still not well understood, and reprogramming of energy metabolism as an additional hallmark of cancer remains unclear [4].

Mitochondria are not only the major energy-producing center via the oxidative phosphorylation (OXPHOS) process coupled with TCA (tricarboxylic acid), but also essential players in the generation of vital cellular metabolites including heme [5] and regulators for cell survival [6]. Mitochondrial homeostasis is tightly controlled to balance mitochondrial fusion, fission, biogenesis, and autophagy. Mitochondrial autophagy (mitophagy) is a selective process of macroautophagy/autophagy targeting mitochondria to protect cells against the release of proapoptotic proteins, the generation of toxic reactive oxygen species (ROS), and the futile hydrolysis of adenosine triphosphate (ATP) by depolarized mitochondria [7]. Under the conditions of mitochondrial membrane potential (ΔΨm) loss and the E3 ubiquitin ligase PARK2/Parkin recruited to degrade several outer mitochondrial membrane (OMM) proteins, PTEN-induced kinase 1 (PINK1) is stabilized onto the OMM to implement the initial mitophagic pathway. The microtubule-associated protein 1A/1B-light chain 3 (LC3) induces aggregation and phagophore nucleation of mitochondria leading to fusion with lysosomes [8]. In general, mitochondria undergo biogenesis and fusion under conditions of increased metabolic demand, while the decreased metabolic requirement may remove the superfluous mitochondria via fission and mitophagy [9]. Autophagy/mitophagy may play a dichotomous role in tumorigenesis to trigger cancer cells to “autophagy-associated cell death” through excessive self-digestion [10] or to support the proliferation and survival of cancer cells by recycling degradation products [11].

The mitochondrion relies on the shuttle of a variety of metabolites and cofactors across the mitochondrial membrane, which is accomplished by a superfamily of 53 membrane-embedded proteins known as the mitochondrial carrier family (MCF) encoded by the human solute carrier (SLC) family 25 genes [12]. As an enigmatic group separate from SLC25, the sideroflexin (SFXN) family is categorized under SLC56 owing to their potential 4 to 5 transmembrane domains (TMDs) [13, 14]. There are five mammalian SFXN (SFXN1–SFXN5) subfamily members with different expression patterns. Among them, SFXN1 was originally identified from fexed-tail (f/f) mice that exhibited sideroblastic-like anemia characterized by excess iron accumulation in mitochondria of erythrocytes [13, 15]. A recent study indicated that SFXN1 directly transported serine into mitochondria for one-carbon metabolism [16]. SFXN2 was also recently reported to participate in mitochondrial iron metabolism and regulate heme biosynthesis in *SFXN2*-knockout cell model [17]. SFXN3 is a mitochondrial protein enriched in neurons of rodent brains [18]. Mutated SFXN4 protein causes mitochondrial disease and facilitates the assembly of mitochondrial complex I [19]. Characterization of these SFXNs has been attracting more and more attention for their great significance in mitochondrial physiopathology.

In present study, we first found that SFXN2 was elevated in MM patients with poor outcomes based on clinical datasets. Next, we utilized MM cell lines and xenograft mouse model to investigate the functions and related mechanism of SFXN2 in the development of MM. We propose that as an OMM protein, SFXN2 may be involved in iron metabolism and mitophagy suppression via enhancing ATP energy production and anti-oxidative stress in MM.

## Results

### Increased SFXN2 is related to poor outcomes of MM patients and promotes MM cell proliferation

**Fig. 1:**
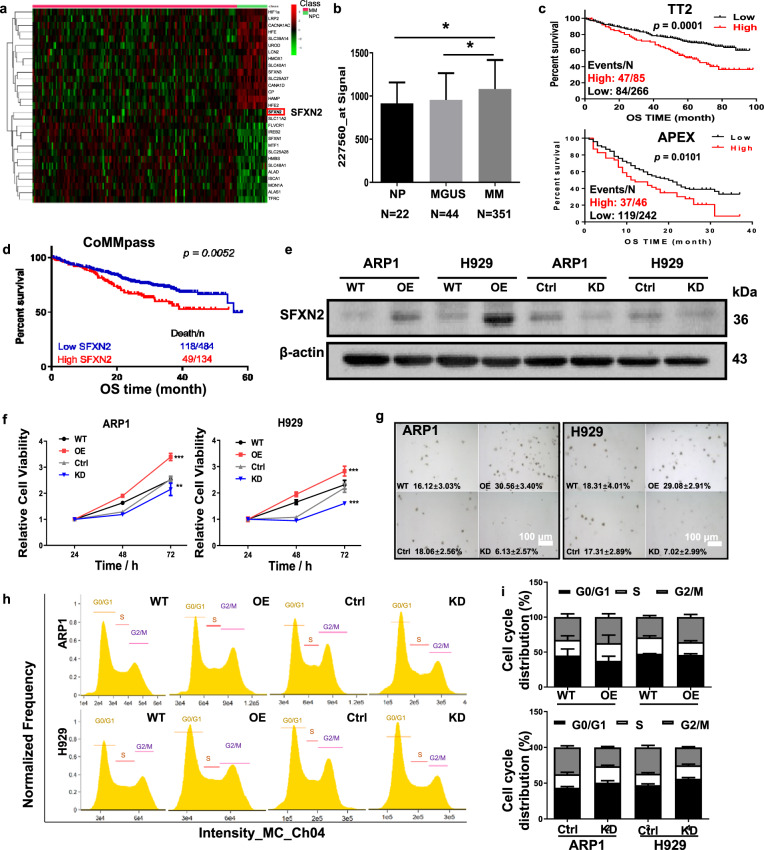
Increased SFXN2 expression is related to poor outcomes of MM patients and high MM cell proliferation. **a** Heatmap showed selective serial genes including SFXN2 from MM GEP cohorts (GSE5900 and GSE2658). **b** Histogram depicted the signal of SFXN2 mRNA (gene antisense probe ID 227560) in different stages of myeloma patients from TT2 cohort. **c** High SFXN2 level was associated with poor overall survival (OS) in newly diagnosed TT2 patients. Reduced OS was presented in recurrent or treatment-resistant patients with high level of SFXN2 from APEX (Assessment of Proteasome Inhibition for Extending Remissions) cohort. **d** RNA-seq datasets from MMREF CoMMpass clinical trial correlated the expression of SFXN2 to higher aggressivity. **e** Validation of SFXN2 overexpression in ARP1 and H929 SFXN2-OE cells relative to vehicle-transfected control cells (Ctrl) and confirmation of SFXN2 knockdown post transfection with three independent SFXN2-targeting shRNAs. **f** Three-day cell growth curve of WT, SFXN2-OE, and SFXN2-KD ARP1 and H929 cells by MTT detection. **g** Representative images of colonies in soft agar formed by SFXN2-OE and SFXN2-KD cells compared to control cells, respectively. **h** Flow cytometry analysis of cell cycle in SFXN2-OE and SFXN2-KD cells compared to control cells, respectively. **i** Quantification analysis showed the proportion of SFXN2-OE and SFXN2-KD cells in G0/G1, S, and G2/M phases relative to control cells, respectively.

Based on a cohort of MM patients from publicly available NCBI Gene Expression Omnibus (GES5900 and GSE2658) (Fig. 1a), we found that SFXN2 mRNA was significantly increased in MM cells compared to normal bone marrow plasma cells (NP) as well as the “premalignant” ones with monoclonal gammopathy of undetermined significance (MGUS) (Fig. 1b). In addition, higher SFXN2 expression was associated with a shorter overall survival (OS) in the newly diagnosed MM cohort from Total Therapy 2 (TT2). In 351 myeloma cases, there were 85 cases at high levels (red curve; 24%) (Fig. 1c). Similar trend was also observed in the recurrent or treatment-resistant patients from APEX cohort (Fig. 1c). In addition, we analyzed MMREF CoMMpass datasets of newly RNA-seq over the course of MM, and found that SFXN2 could be inferred as a proxy of cancer progression from lower to higher aggressivity (Fig. 1d), indicating that exceptionally elevated SFXN2 might predict poor clinical outcomes of MM patients.

To investigate the oncogenic role of SFXN2, we established two human MM cell lines ARP1 and H929, which stably overexpressed SFXN2 cDNA (SFXN2-OE) or inducibly knocked down SFXN2 (SFXN2-KD) based on the lentivirus system (Figs. 1e and S1). The analyses of cell viability and colony formation demonstrated that SFXN2-OE cells proliferated more rapidly than wild-type (WT) in both ARP1 and H929 cells; on the contrary, knockdown of SFXN2 induced by doxycycline (DOX) attenuated cell proliferation and the ability of colony formation compared to their counterparts (Fig. 1f, g). Consistently, the flow cytometric analysis of cell cycle showed an increased number of cells in G2/M phase upon SFXN2 overexpression, while a decreased proportion of G2/M phase cells upon SFXN2 knockdown compared to WT cells (Fig. 1h, i). Therefore, SFXN2 has a significant effect on promoting MM cell proliferation.

### As a mitochondrial outer membrane protein, SFXN2 is involved in bioenergetic processes and stress-induced responses

**Fig. 2:**
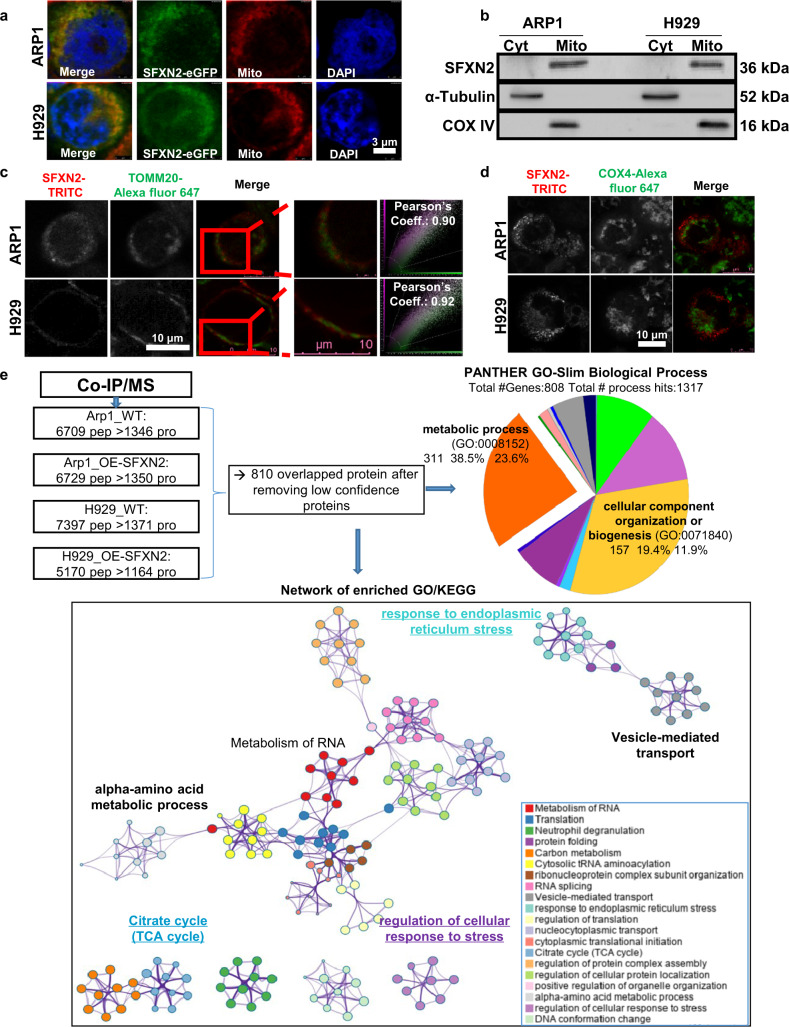
As a mitochondrial outer membrane protein, SFXN2 is involved in bioenergetic processes and stress-induced responses in MM cells. **a** eGFP conjugated SFXN2 (SFXN2-eGFP) was co-localized with the mitochondria-specific MitoTracker Deep Red FM fluorescent dye. **b** WB tested the cytoplasmic and mitochondrial expressions of SFXN2, and the antibodies for α-Tubulin and COX IV were used as loading control, respectively. **c** Immunofluorescence colocation assay showed that a majority of SFXN2-TRITC was co-localized with endogenous TOMM20. **d** The interior membrane of mitochondria lacked SFXN2-TRITC signals. **e** Co-IP/MS identified SFXN2 interacted proteins by using specific antibodies. In total, 810 high and medium confidence proteins were undergone enrichment and network analysis of GO (Gene Ontology) and KEGG (Kyoto Encyclopedia of Genes and Genomes) terms. Pie chart showed # genes, gene hit %, process hits%. Network enrichment of GO/KEGG was generated by using Metascape (metascape.org).

Since SFXN family functions in mitochondrial metabolism, we found the sub-cellular co-localization of SFXN2-eGFP with MitoTracker (Fig. 2a), while the cytoplasmic/mitochondrial separation experiment (Fig. 2b) confirmed its mitochondrial expression. In addition, the SFXN2-TRITC fluorescence signals were highly co-localized with an OMM protein Tomm20 (Fig. 2c), but not co-localized with the interior mitochondria COX4 (Fig. 2d).

In order to further dissect the molecular function of SFXN2, we performed a co-immunoprecipitation combined with Mass Spectrometry (Co-IP/MS) assay using the protein samples of WT and SFXN2-OE ARP1 & H929 cells. In total, we obtained thousands of peptides corresponding to ~810 non-redundant proteins (listed in Table S1). The Gene Ontology (GO) analysis indicated that most of them were classified into metabolic process (GO: 0008152) and cellular component organization or biogenesis (GO: 0071840) (Fig. 2e, upper panel). Interestingly, the network analysis of GO/KEGG terms revealed enrichments of “regulation of cellular response to stress”, “response to endoplasmic reticulum stress”, and “Vesicle-mediated transport”. Therefore, increased SFXN2 might induce the ER stress response possibly via autophagy or mitophagy, since ER and mitochondria shared the phagophore membrane for autophagosome formation [20, 21]. Moreover, the “Citrate cycle (TCA cycle)” and “alpha-amino acid metabolic process” also supported the critical role of SFXN2 in metabolic/bioenergetic processes (Fig. 2e, lower panel). In brief, SFXN2 may control MM cell growth via modulating stress-induced responses, mitophagy, and bioenergetic processes.

### SFXN2 limits starvation-induced autophagy and promotes mitochondrial energy production for MM cell proliferation

**Fig. 3:**
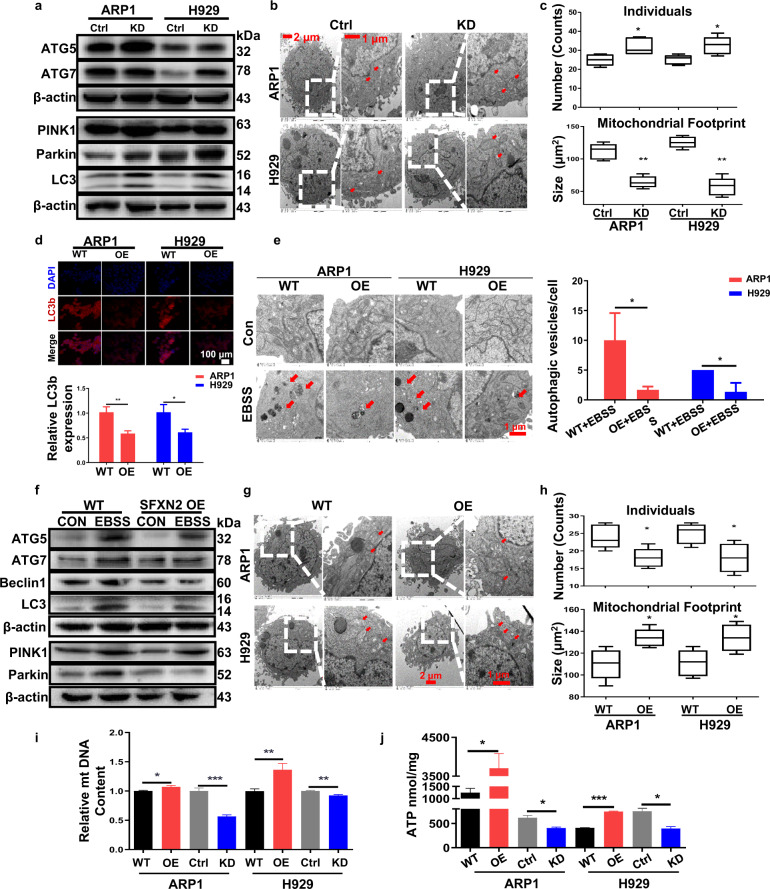
Elevated SFXN2 limits starvation-induced autophagy and promotes energy production for MM cell growth. **a** WB examined the expressions of autophagy/mitophagy-related proteins ATG5, ATG7, LC3, PINK1, and Parkin in Ctrl and SFXN2-KD cells. **b** Representative transmission electron microscopic images of mitochondrial morphology indicated by red arrows in Ctrl and SFXN2-KD cells. **c** Quantification of numbers and sizes of mitochondria by ImageJ in Ctrl and SFXN2-KD cells (*p* < 0.05, using Kruskal Wallis test in R). **d** IF staining of TRITC-labeled LC3b in WT and SFXN2-OE ARP1 and H929 cells. Quantification of fluorescence intensity of TRITC-labeled LC3b in WT and SFXN2-OE ARP1 and H929 cells (*n* = 3 in every group, *p < 0.05, **p < 0.01). **e** Representative photographs showed autophagic vesicles indicated by red arrows in WT and SFXN2-OE ARP1 and H929 cells post EBSS treatment. Scale bars: 0.5 μm. Quantification of starvation-induced autophagic vesicles in WT and SFXN2-OE cells (*n* = 3 in each group, *p < 0.05). **f** WB examined the autophagy-related proteins ATG5, ATG7, Beclin1, LC3, PINK1, and Parkin in EBSS-treated WT and SFXN2-OE ARP1 cells compared to non-treated cells. **g** Transmission electron microscope testified representative mitochondrial morphology indicated by red arrows in WT and SFXN2-OE cells. **h** Numbers and sizes of mitochondria were analyzed by ImageJ (*p* < 0.05, using Kruskal Wallis test in R). **i** Relative mtDNA content of WT, SFXN2-OE, SFXN2-Ctrl, and SFXN2-KD cells were measured by a competitive PCR method. **j** The level of ATP in WT, SFXN2-OE, SFXN2-Ctrl, and SFXN2-KD cells were determined by the ATP Bioluminescence Assay Kit (*p < 0.05, ***p < 0.001).

Following the above conception, we further explored the association between SFXN2, autophagy/mitophagy and mitochondrial energy production. We observed remarkable increase of autophagy-related genes ATG7 and ATG5 upon SFXN2 knockdown in MM cells (Figs. 3a and S2). Of note, WB analysis confirmed that the expressions of PINK1 and Parkin were increased in SFXN2-KD cells after induced by DOX for 48 h (Figs. 3a and S2). PINK1 and Parkin-dependent mitophagy was directly evidenced by the abnormal ultrastructure of individual mitochondria including punctate, rods, and large/round structures and smaller mitochondrial footprints/area upon SFXN2 knockdown (Fig. 3b, c). Generally, autophagy is enhanced under the stressed condition of nutrition-depletion. We checked the effect of SFXN2 on LC3 conversion and other autophagy indicators in MM cells cultured with Earle’s Balanced Salt Solution (EBSS). Upon starved for 48 h, high rates of autophagy/mitophagy were induced in MM cells, which were indicated by the immunofluorescent (IF) staining of LC3 (Fig. 3d). Consistently, the morphological of autophagic vesicles (autophagosome and autolysosome) per cell under transmission electron microscope verified that increased SFXN2 could limit starvation-induced autophagy (Fig. 3e). Meanwhile, elevated SFXN2 significantly alleviated EBSS-induced autophagy that was evidenced by WB analysis for autophagy-related markers ATG5/7 and Beclin1, and mitophagy-related markers PINK1 and Parkin at protein level (Figs. 3f and S3) and mRNA level (Fig. S4). We also examined the ultrastructure of mitochondria in SFXN2-OE cells (Fig. 3g). In contrast, there were less mitochondria individuals and bigger mitochondrial footprints in SFXN2-OE cells compared to WT cells (Fig. 3h), suggesting that the reduced fragmentation and frequent fusion network was associated with SFXN2-suppressed mitophagy. The mtDNA content, partially reflecting the cellular proliferation status, was significantly increased in SFXN2-OE cells while decreased in SFXN2-KD cells compared to control cells, respectively (Fig. 3i). In addition, we found that some mtDNA-encoded genes required for ATP synthesis were de-regulated upon SFXN2 overexpression or knockdown, including cytochrome C oxidase I (COX1) and cytochrome b-c1 (cytb) (Fig. S5). Then, we detected the bioenergy production in both SFXN2-OE and SFXN2-KD cells. As Fig. 3j shown, there was more production of net ATP in SFXN2-OE cells while less ATP in SFXN2-KD cells compared to control cells. Collectively, SFXN2 promotes MM cell proliferation via regulating mitochondrial autophagy and energy production.

### SFXN2 modulates bioenergetic processes via accelerating cellular iron utilization and increases tumor burden in MM xenograft model

**Fig. 4:**
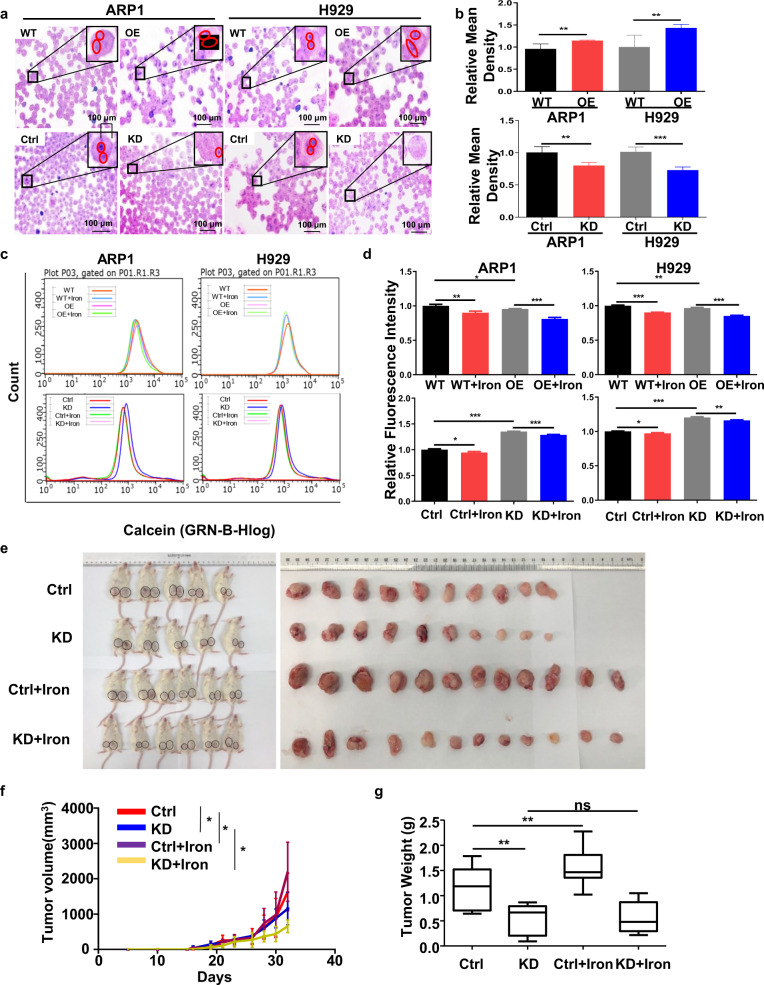
SFXN2 modulates bioenergetic processes via accelerating cellular iron utilization and aggravates tumor burden in MM xenograft model. **a** The cellular iron contents of WT, SFXN2-OE, SFXN2-Ctrl, and SFXN2-KD cells were examined by using Prussian blue iron staining. **b** Quantification of Prussian blue iron staining. **c** Flow cytometry analysis of ferrous ion content indicated by Calcein-AM fluorescence intensity. **d** The iron content was significantly higher in SFXN2-OE cells than WT cells while the iron content in SFXN2-KD cells was lower than control cells. **e** The images of the xenograft mice and xenografts taken from the mice. **f** The analysis of tumor volume. **g** The analysis of tumor weight.

Recently, Mon EE et al. reported the role of SFXN2 in mitochondrial iron homeostasis [17]. We tested the total cellular ferric (Fe^3+^) iron content using Perls Prussian blue staining in MM cells (Fig. 4a). The quantification analysis showed that SFXN2 was positively associated with iron content (Fig. 4b). The fluorescent Calcein-AM probes (Fig. 4c) were applied to quantify the chelatable cytosolic Fe^2+^ by the quenching of fluorescent Calcein signal. As Fig. 4d shown, the fluorescence intensity was weaker in SFXN2-OE cells than that in WT cells, indicating that chelatable cytosolic Fe^2+^ was increased; on the contrary, decreased cytosolic Fe^2+^ was observed in SFXN2-KD cells compared with control cells (Fig. 4d).

To extend the therapeutic potential of SFXN2 in iron metabolism in vivo, we established MM xenograft mouse model. The NOD-SCID mice subcutaneously injected SFXN2-KD ARP1 cells were divided into four groups, and the xenografts were shown in Fig. 4e. Tumor growth curve displayed that the tumors in control mice grew faster than SFXN2-KD mice no matter they were treated with Iron Dextran or not, while the tumor volume of SFXN2-KD mice treated with Iron Dextran was significantly decreased compared with non-treated SFXN2-KD mice (Fig. 4f). The analysis of tumor weight showed that the tumors in SFXN2-KD mice were much lower than in control mice treated with Iron Dextran (Fig. 4g). Therefore, inhibition of SFXN2 significantly suppresses MM cell growth in vivo under the condition of iron supplement.

### SFXN2 controls iron-induced oxidative stresses as well as mitochondrial heme biosynthesis

**Fig. 5:**
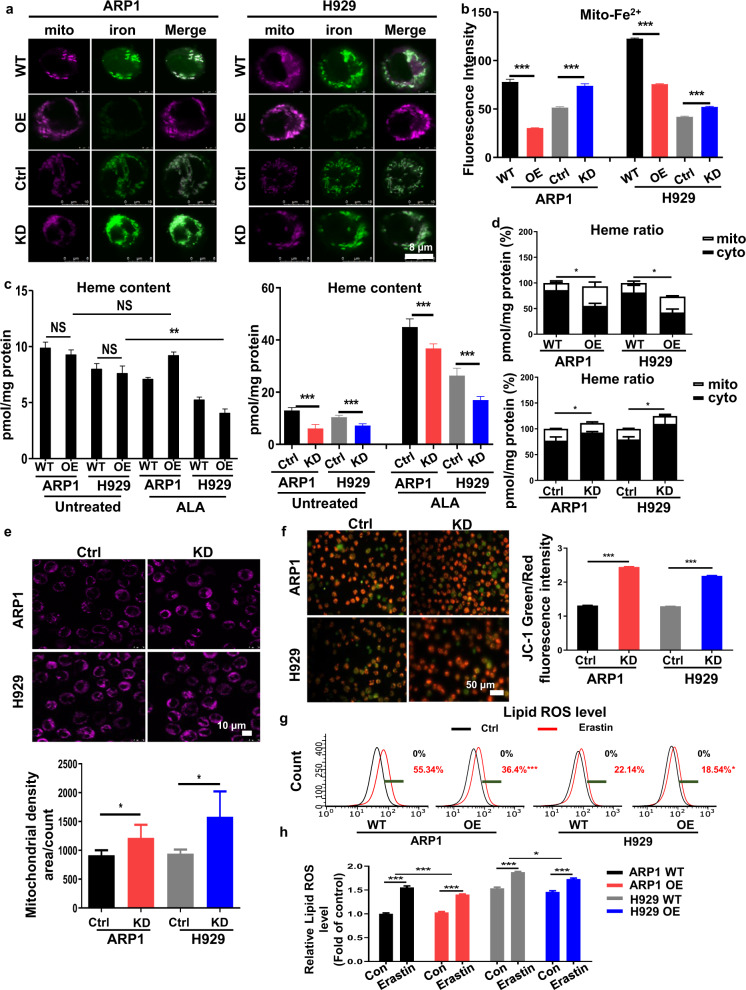
SFXN2 controls iron-induced oxidative stresses as well as mitochondrial heme biosynthesis. **a** The fluorescent probes labeling mitochondria ferrous iron were applied in WT, SFXN2-OE, SFXN2-Ctrl, and SFXN2-KD cells individually. **b** Quantification analysis of fluorescence intensity. **c** The labile heme content in MM cells with or without the treatment of extra 5-Aminolevulinic acid (5-ALA). **d** The heme content showed a majority of heme in mitochondria with altered ratio of cytoplasm mitochondrial heme content. **e** Fluorescence intensity of mitochondria in Ctrl and SFXN2-KD cells (upper panel) and quantification analysis of fluorescence intensity (lower panel). **f** Variations in mitochondrial membrane potential (Δψm) in MM cells were tested by using a JC-1 dye (left panel). Quantification analysis of Δψm were performed in Ctrl and SFXN2-KD cells (right panel). **g** Flow cytometry analysis showed intracellular lipid ROS levels in MM cells treated with ferroptosis/ROS inducer Erastin. **h** Intracellular lipid ROS levels were quantified through the fluorescence intensity of BODIPY probe.

Since most of the cellular iron is utilized as essential cofactors of mitochondrial respiration chain enzymes [22], we detected the mitochondria localized ferrous (Fe^2+^) iron using specific fluorescent probes. The fluorescence intensity was significantly higher in SFXN2-KD cells than control cells, however, this trend was opposite in SFXN2-OE cells (Fig. 5a, b). Next, we measured the labile heme content, as the labile heme content was in proportion to the total heme content [23]. There was no significant difference between WT and SFXN2-OE cells, though SFXN2-OE ARP1 cells displayed a significant increased heme when supplied with extra 5-Aminolevulinic acid (5-ALA) as precursor intermediate for heme biosynthesis (Fig. 5c, left panel). In contrast, the labile heme content was much lower in SFXN2-KD cells than control cells, even supplied with 5-ALA (Fig. 5c, right panel). In addition, we observed a decrease or increase of mitochondrial/cytoplasmic heme ratio (percentage) upon SFXN2 overexpression or knockdown, respectively (Fig. 5d). These results suggest that SFXN2 enhances mitochondrial iron utilization and turnover to favor the energy production and growth of MM cells.

Iron usage may cause extra ROS as by-product during energy production [24]. A well-recognized tight coupling is between mitophagy activation and ROS production [25, 26]. Thus, we were motivated to investigate the relationship between SFXN2-mediated mitophagy and iron-induced ROS stress. The mitochondria staining results showed that higher fluorescence density was presented in SFXN2-KD cells induced by DOX for 48 h than control cells (Fig. 5e), indicating a frequent mitochondria fission during mitophagy. Since the mitochondrial membrane potential (ΔΨm) enables the initiation of the PINK1/PARK-dependent mitophagy [27], we detected the ΔΨm using JC-1 probe, a potential-sensitive dual-emission dye (Green-fluorescent as monomer at low potential) aggregating in functional mitochondria with high ΔΨm (Red). As shown in Fig. 5f, the significant increased JC-1 Green/Red intensity ratio post SFXN2 knockdown confirmed the critical function of SFXN2 in maintaining ΔΨm. High utilization and turnover of iron in SFXN2-OE cells may increase the demand of more ROS generation as by-product. We measured the intracellular lipid ROS level in MM cells using a lipid-soluble ratiometric fluorescent sensor BODIPY™ 581/591 C11 (Fig. 5g, h). However, no significant difference was observed between WT and SFXN2-OE cells (Fig. 5h). Erastin is a ferroptosis activator that induces iron-dependent ferroptosis and triggers cytosolic ROS accumulation [28]. When Erastin was employed to treat WT and SFXN2-OE cells, lower ROS levels were observed in SFXN2-OE cells than WT cells (Fig. 5h). Meanwhile, the treatment of glutathione (GSH) biosynthesis inhibitor BSO (L-buthionine-S, R-sulfoximine) enabled SFXN2-OE cells to produce more ROS than WT cells (Fig. S6). Through silencing SFNX2 by siRNA, the ROS production was suppressed while the expressions of autophagy/mitophagy-related proteins ATG7, ATG5, LC3, and Parkin, PINK1 were increased (Fig. S7a–d). We assume that SFXN2 may control iron-induced oxidative stress to keep autophagy at certain level, which will shed light on a mechanism of enhancing the tolerance of ROS-induced cytotoxicity in MM cells.

### SFXN2 alleviates mitophagy and ROS by interacting with HO1 in iron metabolism pathway

**Fig. 6:**
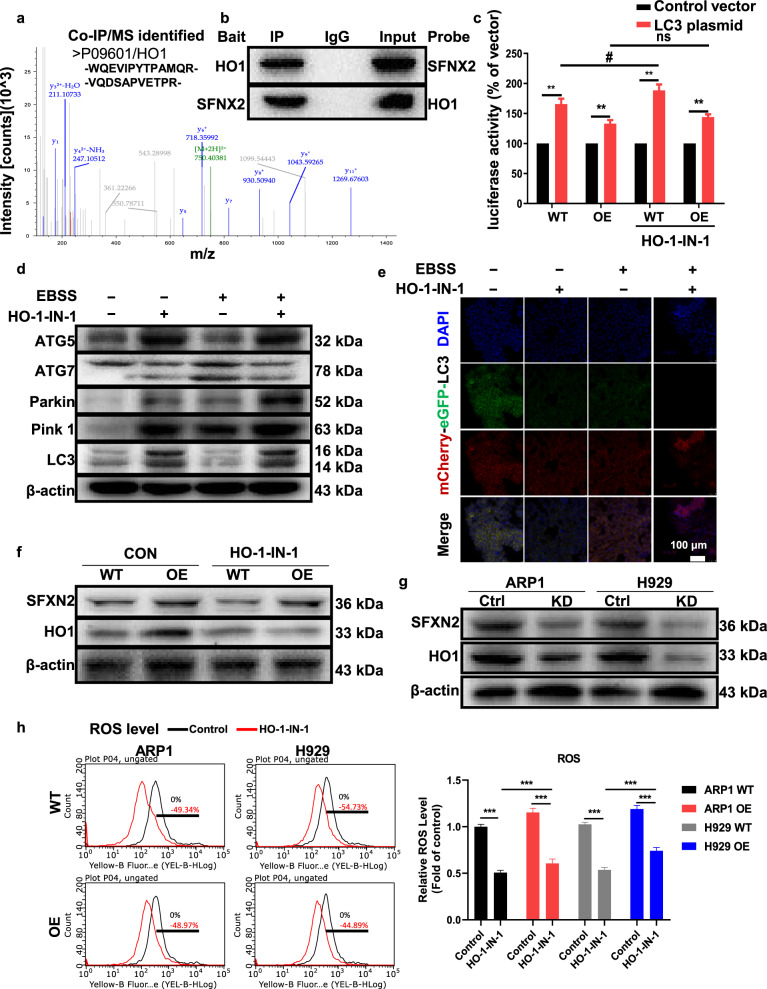
HO1-mediated anti-oxidant effect contributes to SFXN2-suppressed mitophagy and ROS production during iron metabolism. **a** Co-IP/MS identified two original peptide sequences corresponding to HO1 (unipro ID: P09601). **b** IP assay showed the interaction between SFXN2 and HO1. **c** Luciferase activity driven by a LC3B promoter sequence screened potent molecules influencing autophagic activity in WT and SFXN2-OE ARP1 cells before and after treatment of HO1 inhibitor (HO-1-IN-1). **d** WB analysis showed the expressions of mitophagy-related proteins ATG7, ATG5, Parkin, PINK1, and LC3 in ARP1 cells treated with EBSS and HO-1-IN-1 individually or both. **e** ARP1 cells transduced with mCherry-eGFP-LC3 were incubated with either complete media or EBSS for 48 h. All the images were captured with the confocal microscope (mCherry-LC3, Red; eGFP-LC3, Green). **f**, **g** WB tested the expressions of HO1 and SFXN2 in SFXN2-OE (**f**) and SFXN2-KD (**g**) cells. **h** Flow cytometry analysis examined intracellular ROS levels in WT and SFXN2-OE cells treated with HO-1-IN-1(left panel). Quantification analysis for fluorescence intensity was performed in WT and SFXN2-OE cells (right panel).

It is of great importance to identify the downstream targets/pathways involved in SFXN2-mediated mitophagy with anti-oxidative response. We focused on the Heme oxygenase 1 (HO1, encoded by HMOX1 gene) relying on two reasons: on the one hand, HO1 physically interacting with SFXN2 was validated by the analyses of Co-IP/MS (Fig. 6a) and WB (Fig. 6b); on the other hand, HO1 participated in SFXN2-mediated autophagy according to a screen of LC3B promoter driven pGL3-Luciferase reporter system. As shown in Fig. 6c, the treatment of HO1-specific inhibitor HO-1-IN-1 with an IC_50_ of 250 nM significantly improved the luciferase activity in WT cells but not in SFXN2-OE cells compared to mock treatment, this might be due to the relative suppressed mitophagy in SFXN2-OE cells.

The expressions of autophagy/mitophagy-related proteins ATG7, ATG5, Parkin, PINK1, and LC3 were tested in ARP1 cells treated with HO-1-IN-1 and EBSS individually or both. HO-1-IN-1 treatment resulted in significant elevation of these proteins and LC3 conversion, and the activation effect was even stronger than EBSS starvation to a certain extent (Fig. 6d). Moreover, inhibition of HO1 alleviated the suppression on ATG7, ATG5, LC3, and PINK1 in SFXN2-OE cells (Fig. S8). We monitored the autophagic flux using the tandem tagged mCherry-eGFP-LC3 probe to detect autophagosomes (mCherry + eGFP+; yellow) and autolysosomes (mCherry+; red puncta) based on pH-sensitive eGFP in lysosomes. Consistently, inhibition of HO1 enhanced the autolysosomes formation especially coupled with EBSS starvation (Fig. 6e). WB analysis confirmed that HO1 was increased in SFXN2-OE cells while decreased in SFXN2-KD cells compared to control cells, respectively (Figs. 6f, g and S9).

We also checked the effect of HO-1-IN-1 on intracellular ROS production. The HO-1-IN-1 treatment triggered less ROS production by ~50% in both WT and SFXN2-OE cells than non-treated control cells, and SFXN2-OE cells were prone to release more ROS than WT cells (Fig. 6h). These results suggest that SFXN2 adjusts iron-mediated energy production together with HO1 to decrease mitophagy and ROS generation.

## Discussion

**Fig. 7:**
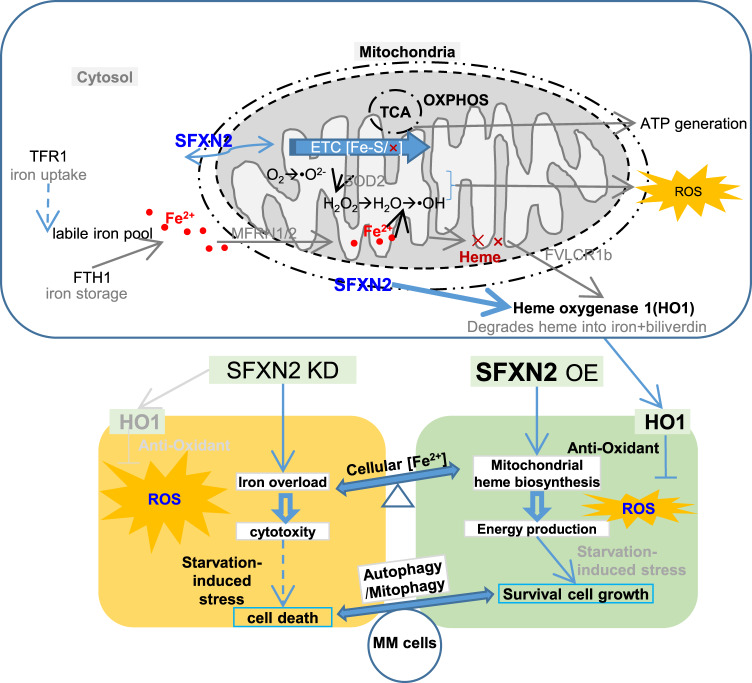
Schematic working model shows the oncogenic role of SFXN2 in alleviating mitochondrion autophagy while enhancing energy production and HO1-mediated anti-oxidative effect in MM cells. The upper panel shows that SFXN2 is involved in mitochondrial heme biosynthesis for ATP production as well as the by-product ROS generation. Cytosol iron acquired from TFR1 is either stored in FTH1 or released as labile iron pool (LIP). Then it is exported by a mitochondrial heme exporter FVLCR1b identified in erythrocytes to transfer into mitochondria by MFRN1/2 for mitochondrial ATP synthesis during ETC couples with TCA and OXPHOS. SFXN2 regulates mitochondrial iron utilization and anti-oxidative stress together with HO1. The lower panel indicates that SFXN2 promotes MM cell proliferation through maintaining the homeostasis among mitochondrial bioenergetics, autophagy, iron metabolism, and redox in an interconnected way. Color or bold letters present molecules/processes in this study, and gray letters indicate molecules/processes in other research work. Solid arrows mean direct interaction, and dashed lines or arrows mean indirect effects.

The present study determined the oncogenic role of SFXN2 during MM tumorigenesis and revealed the therapeutic potential of targeting SFXN2 related to iron metabolism. Elevated SFXN2 was associated with poor outcomes of MM patients (Fig. 1a–d), and anti-myeloma effect of SFXN2 knockdown was evidenced in xenograft model (Fig. 4e–g). Intriguingly, the interdependency between SFXN2 and clinical outcomes also exists in Acute Myeloid Leukemia (AML) based on the gene expression profiling data from the TCGA (http://gepia.cancer-pku.cn/detail.php?gene=sfxn2) [29]. As an OMM protein, SFXN2 played multiple-functional roles in regulating mitochondrial bioenergetics, autophagy, iron metabolism, and redox homeostasis in an interconnected and intricate way (Fig. 7).

SFXN2 controlled iron metabolism (Figs. 4 and 5) and ATP production (Fig. 3j) during metabolic rewiring in MM cells. In mammals, the five SFXN homologs with distinct expression-patterns have diverse functions related to mitochondria homeostasis, largely depending on the shuttle of many metabolites across the mitochondrial membranes [12]. Although the exact substrate specificities of SFXNs are required to be further dissected, it is undoubted that SFXN2 plays a critical role in mitochondrial homeostasis and bioenergy production. Since the discovery of Warburg effect in the 1920s, evidence has been mounting that hyperplastic tumor tissues are more dependent on glycolysis even in aerobic conditions [30], and altered bioenergetics or metabolic reprogramming are emerging hallmarks of cancers to fuel the increased energy demand. However, increasing reports show that most tumor cells maintain normal mitochondrial fitness and intact OXPHOS respiration to generate ATP [31–33]. Generally, tumor cells lack OXPHOS-to-glycolysis switch but rather drastically increase glycolysis. It is a strong support for our work that metabolic reprogramming is not only for ATP generation but also for reducing equivalent biomass synthesis including NADPH to balance ROS or other oxidative stresses [32].

Autophagy is induced by various cellular stresses and increasingly recognized as double-edged player during tumorigenesis [34]. Basal autophagy may protect cells from excessive free radical damage and genetic instability, thus preventing malignant transformation [35, 36]. By contrast, autophagy prevents tumor cells from apoptosis to support tumor growth [37]. Our exploration on autophagy-related factors, autophagic vesicles, and flux reflected that SFXN2 suppressed starvation-induced autophagy via influencing ΔΨm and mitophagy in a PINK1/PARK-dependent manner (Figs. 3a, f, 5e, and 6d). As a hematological malignancy, MM bears a high level of basal autophagy compared to other solid tumors. It is plausible that B cell-derived MM cells keep synthesizing/secreting large amounts of “M” protein, which make them sustain enlarged ER stress and more autophagy or energy demands for survival [38]. ER extensively interacts with mitochondria by sharing the phagophore membrane for autophagosome formation [20, 39], thus we speculated that MM cells resisted the ER stress by limiting mitophagy (Fig. 3). In agreement with above conception, some proteins potentially interacting with SFXN2 were classified into GO/KEGG terms of cellular ER-mediated stress and bioenergy metabolic process (Fig. 2e). Our RNA-seq analysis in SFXN2-OE ARP1 and H929 cells compared to WT cells (data not shown) also showed similar terms, such as “regulation of cellular response to stress (GO:0080135)”, “cellular macromolecule biosynthetic process (GO: 0034645)”, and “negative regulation of catabolic process (GO: 0009895)”.

The intricate relationships among cellular redox homeostasis, autophagy, and cell growth are complicated, while iron is an essential factor in the mitochondrial bioenergetics. First, iron is a requisite nutrition for almost all critical physiological and cellular functions, especially for efficient bioenergy production as elemental heme [40]. Second, dietary or cellular iron has a double nature in relation to tumorigenesis. Either iron deficiency or over sufficiency may cause cellular stresses, thus altering the autophagic status even cell death [41, 42]. Cancer cells develop a dependence on iron well over that of their non-malignant counterparts, which is termed as “iron addiction” [43]. Consistently, the supplement of iron at supra-physiological level indeed showed a carcinogenic effect on MM model mice (Fig. 4e). Third, the effects of SFXN2 on promoting MM cell proliferation and suppressing autophagy might be secondary to mitotoxicity due to iron accumulation in the mitochondria and partially bearing on ferritinophagy or even ferroptosis (data not shown). Ferroptosis is a recently defined iron-dependent form of non-apoptotic cell death by Dr. Stockwell and colleagues [28]. SFXN1-dependant iron overload mediates ferritinophagy activation in cardiomyocytes hypertrophy [44]. Fourth, the cellular iron level is tightly balanced to avoid excessive ROS during the Electron Transport Chain (ETC) based on redox cycle to gain and lose electrons, such as the Fenton and Haber-Weiss reactions [45, 46].

To balance the Fe^2+^-catalyzed ROS by-product from SFXN2-mediated iron turnover in MM, HO1 was recruited by elevated SFXN2 to execute anti-oxidant activity (Figs. 5, 6, and S4), possibly depending on the moderate but not excessive activated level of HO1 expression [47, 48]. HO1 is also the rate-limiting enzyme in heme catabolism by degrading heme into iron, carbon monoxide and the endogenous antioxidants biliverdin/bilirubin [49], which has a cytoprotective role to conquer the oxidative stress induced by chemotherapeutic agents in tumor cells, thus preventing the cancer cells from apoptosis and autophagy [50, 51]. Interestingly, HO1 is reported to be involved in Bortezomib-induced drug-resistance and cellular proliferation in MM [52, 53]. Though it is emerging as a novel therapeutic target in hematological malignancies [54, 55], the effect of HO1 inhibitor on ROS production (Fig. 6h) needs to be noticed partially as a result of detrimental effect caused by heme metabolites or iron accumulation [50, 53, 56]. There are substantial and growing evidences of targeting iron metabolism to develop the treatment for various cancers based on the metabolic vulnerability of iron deficiency or excess [42]. Taking advantage of the balance between the cell type/status-specific energy demand and cytotoxic oxidative stresses [57, 58] to develop mitochondrial-targeting anticancer drugs, antioxidants and sensor molecules [59] still requires more intensive investigation.

Taken together, the present study demonstrates that SFXN2 promotes MM cell proliferation via suppressing PINK1/PARK mediated mitophagy and HO1-mediated anti-oxidative stress in concert and intersect with iron metabolism. Collectively, our work provides new insights into SFXN2-mediated mitochondrial homeostasis and bioenergy production, and reveals that targeting SFXN2 may be a promising strategy for the treatment of MM patients.

## Materials and methods

### Cell culture

Human ARP1 and H929 cells were kind gifts from Dr. Siegfried Janz (University of Iowa, Iowa City, IA, USA). Cells were cultured in RPMI-1640 medium (Biological Industries, Beit Haemek, Israel) supplemented with 10% heat-inactivated fetal bovine serum (FBS; Biological Industries, Israel) and 1% penicillin/streptomycin at 37 °C with 5% CO_2_ mycoplasma-free condition was secured before further experiments.

### Antibodies and reagents

HO1 inhibitor HO-1-IN-1 hydrochloride was purchased from MedChemExpress. The following commercial antibodies were used in this study: SFXN2 (ab67191), TOMM20 (ab186734) from Abcam; HO1 (66743-1-Ig), PINK-1(23274-1-AP), PARKIN (14060-1-AP), and P62/SQSTM1 (18420-1-AP) from ProteinTech; Atg7 (#2631s), Beclin-1 (#3738), LC3A/B (#12741), α-tubulin (#2125s), β-actin (#3700s), and goat anti-rabbit IgG-HRP (#7074) from Cell Signaling Technology; Atg5 (Biological, 110-53818), COX4 (Bioss, bsm-33037M), goat anti-mouse IgG-HRP (SANTA, SC-2005).

### Cell proliferation assay, cell cycle, and colony formation assay

Cell proliferation was evaluated as described previously [60]. For MTT assay, a density of 8 × 10^3^ MM cells/well in 96-well plate was cultured for 48 h. For cell cycle assay, 1 × 10^6^ cells were washed twice with PBS, fixed with 75% ethanol for 12 h, treated with 200 μg/mL RNase for 15 min, and stained with 50 μg/mL propidium iodide (PI) (Yeasen, China) before analyzed by FlowSight flow cytometer (Merck Millipore, Germany). For colony formation assay, 1 × 10^4^ cells in 0.5 mL of 0.33% agar/RPMI1640 supplemented with 10% FBS in 12-well plate were fed twice/week for 2 weeks. Cell clusters were considered to be a colony if >40 cells were present. The colonies were imaged by a microscope, and colony numbers were counted by using ImageJ software. The data of colony numbers represent mean ± SD from at least three independent experiments.

### Western blot and RT–qPCR

Detailed western blot and RT–qPCR procedures were described in our previous report [60]. All reactions were performed as triplicates. The primers used in this study were shown in Table S2.

### Determination of Δψm

Mitochondrial Deep Red fluorescent probe MitoTracker Deep Red FM was purchased from Yeasen Biotechnology Co., Ltd. (Shanghai, China, NO. 40743ES50). Variations in mitochondrial membrane potential (Δψm) were measured using a JC-1 kit (Beyotime Institute of Biotechnology, Jiangsu, China, #C2006). After treated with EBSS, a total of 2 × 10^6^ cells were harvested and incubated with JC-1 at 37 °C for 20 min and then washed and resuspended in PBS. The samples were analyzed and 10,000 events were acquired with flow cytometer.

### Mitochondrial isolation assay

Mitochondria isolation was operated according to the manufacturer’s instruction of Cell Mitochondria Isolation Kit (Beyotime Institute of Biotechnology, Jiangsu, China, #C3601). 5 × 10^8^ ARP1 and H929 cells were treated with EBSS or RPMI-1640 medium for 48 h. After treatment, cells were washed with cold PBS for three times, then resuspended with isolation buffer containing protease inhibitor (1:1000, Biolegend, America, CAT: 640941). After standing the suspension for 15 min, the cells were homogenized by a Dounce glass homogenizer for several complete up-and-down cycles and kept on ice. Next, the liquid was centrifuged to remove debris at 600 × *g* for 10 min at 4 °C. Then supernatant centrifuged at 11,000 × *g* for 10 min at 4 °C. The pellet was the crude mitochondrial fraction and lysed by mitochondrial lysis fluid for western blot.

### Perls Prussian blue iron staining

Perls staining stains iron in blue and other tissues in red. Cells attached to the slides were fixed in 10% neutral buffered formalin for 10 min, washed and stained with Perls’ stains at 56 °C for 4 h. Then the slides were stained with nuclear fast red at room temperature for 1 min and imaged using Image-pro Plus 6.0 (Media Cybernetics, Inc).

### Measurement of heme concentration

Intracellular heme concentration was measured by a porphyrin fluorescence assay [61]. Total 1 × 10^5^ MM cells were resuspended in 0.5 mL of 2 M oxalic acid and heated at 100 °C for 30 min. Standard solutions of protoporphyrin with the concentrations of 0, 0.01, 0.1, 1, 10, and 100 nM were prepared with oxalic acid. Read fluorescence of porphyrin using 400 nm excitation and 662 or 608 nm emission. The relative heme ratio was calculated based on the absolute meme content of mitochondrial/cytoplasmic heme ratio (percentage).

### Measurement of Fe^2+^ levels

MM cells (1 × 10^6^) were harvested and incubated with 5 μM of Calcein-AM (a nonfluorescent lipophilic ester, Yeasen, Shanghai, China, #40719ES50) for 15 min at 37 °C and 50 μg/mL PI for 10 min at 4 °C after treated with EBSS for 48 h. Then the cells were washed twice with PBS and the fluorescence intensity signals of the cells were analyzed by flow cytometry. The reduction of Calcein-AM fluorescence intensity represented an increase of chelatable cytosolic Fe^2+^. Mitochondrial ferrous iron (Fe^2+^) fluorescent probe Mito-FerroGreen was purchased from DojinDo (NO. M489).

### ROS determination

Intracellular ROS levels were determined as described previously [62] and quantified by measuring the fluorescent BODIPY 581/591 C11 (Invitrogen; USA; #D3861) or the 2’,7’-dichlorofluorescein diacetate (DCFH-DA; Beyotime, China, #S0033) probes by flow cytometry.

### Determination of ATP and mtDNA content

The levels of ATP were determined by using an ATP Bioluminescence Assay Kit (#S0026, Beyotime, China). Total DNA was extracted by using a bioluminescence kit (D0061, Beyotime, China) and mtDNA copy number was presented as a ratio of COX1 to 18S rDNA based on TaqMan PCR as previously described [62].

### Lentivirus plasmids and transfection

Lentiviruses were produced by co-transfection of the expression vector of interest with the packaging plasmids PLP1, PLP2, and VSVG into HEK293T cells using Lipofectamine™ 2000 Transfection Reagent (Invitrogen, USA). Virus supernatant was collected after 48 h. Transfected MM cells were selected by puromycin to obtain a stable and heterogenous population of puromycin-resistant cells. Transduction efficiency was determined by WB test. Plasmids containing human SFXN2 cDNA (NM_178858) and SFXN2 shRNA cassettes were purchased from Generay Biotech Co., China. The SFXN2 coding sequence fused with Flag was cloned into the lentiviral vector, CD513B-1. SFXN2-targeting shRNA under the control of a DOX-inducible promoter was cloned into the pTRIPZ vector. Three synthetic siRNAs were also purchased from GenePharma (Shanghai, China). Detailed RNAi sequences were listed in Table S2.

### Immunofluorescent staining

After fixation and permeabilization, cells were incubated with primary antibodies (LC3-II, Abcam, #48394; SFXN2, Abcam, #67191) at 4 °C overnight and secondary goat anti-rabbit IgG/TRITC (Abcam, ab6718) or goat anti-mouse IgG/TRITC (Abcam, #6786), respectively. For autophagy experiments, cells transduced with mCherry-eGFP-LC3 were starved in EBSS for 48 h. All images were captured by confocal microscope (TCS SP8; Leica, Germany).

### Transmission electron microscope

The samples were prepared as described previously [63]. Images were taken under the Olympus EM208S transmission electron microscope. We counted the numbers and calculated the sizes of mitochondria by ImageJ [64].

### Luciferase reporter system

The human *LC3B* promoter region (−1000, +200 bp) was subcloned into pGL3-Luciferase (Promega) to construct the chimeric pGL3-*LC3B*-Luc-3’UTR. Transfection efficiency was normalized to phRL-null control reporter (Promega). Luciferase activity was measured by Dual Luciferase Assay System (Promega).

### Mass spectrometry analysis

In order to screen the potential interacting proteins and pathways, we performed the Co-IP experiment per the manufacture’s instruction (Thermo scientific Pierce™ Direct Magnetic IP/Co-IP Kit, Catalog number: 88828) using SFXN2 specific antibody (Abcam, ab67191) and IgG as negative control in WT and SFXN2-OE ARP1 and H929 cells. SDS-PAGE was used to separate proteins, and gel bands were excised and digested with trypsin (Promega, USA). The resulting peptides were analyzed by using a QExactive mass spectrometer (Thermo Fisher Scientific). The downstream bioinformatics analysis of GO was conducted at http://geneontology.org/, and the enriched GO/KEGG term data-mining took advantage of an online tool MetaScape at https://metascape.org/. The proteomics data were deposited to the ProteomeXchange Consortium (http://proteomecentral.proteomexchange.org) via the iProX partner repository with identifier PXD027607.

### Human myeloma xenograft mouse model

All healthy 6–8 weeks old NOD-SCID mice with similar body weight were randomized blindly into control or treatment groups. ARP1 SFXN2-KD cells (2 × 10^6^) were subcutaneously injected into NOD-SCID mice (*n* = 5 or 6 per group) from Beijing Vital River Laboratory Animal Technology (Beijing, China). The mice were treated with DOX (2 mg/mL) or saline solution every other day. The iron supplement with dose of 40 mg iron-dextran/kg was injected intraperitoneally as previously described [65]. Once the tumor diameter reached 20 mm, the mice would be sacrificed. All animal procedures were conducted following government-published recommendations for the Care and Use of Laboratory Animal, and approved by the Institutional Ethics Review Boards of Nanjing University of Chinese Medicine (Nos. ACU170501 and 201905A003).

### Statistical analysis

All data were shown as mean ± SD for ≥3 independent experiments. Differences between groups were determined using two-sided Student’s *t*-test or one-way ANOVA. The survival data were calculated by the Kaplan–Meier method and analyzed by log-rank (Mantel–Cox) test. The statistical significance was set at *p < 0.05, **p < 0.01, and ***p < 0.001 using GraphPad Prism 5 software (GraphPad Software Inc., USA).

## Supplementary information

Supplementary Figures S1-S9

Table S1 list of possible SFXN2-interacting proteins identified by CoIP-MS

Table S2 oligo sequences used in this study

Original Data File

aj-checklist

## Data Availability

The proteomics data were deposited to the ProteomeXchange Consortium (http://proteomecentral.proteomexchange.org) via the iProX partner repository with identifier PXD027607. All data that support the findings of this study are available from the corresponding authors upon reasonable request.
