# Supplementary material for: Elevated SFXN2 limits mitochondrial autophagy and increases iron-mediated energy production to promote multiple myeloma cell proliferation
Source: Cell Death Dis. 2022 Sep 26;13(9):822. doi: 10.1038/s41419-022-05272-z (PMC9513108; doi:10.1038/s41419-022-05272-z)
Supplement: Supplementary file 1 — Supplementary Figures S1-S9 [file 41419_2022_5272_MOESM1_ESM.doc]

**Supplementary Figures**


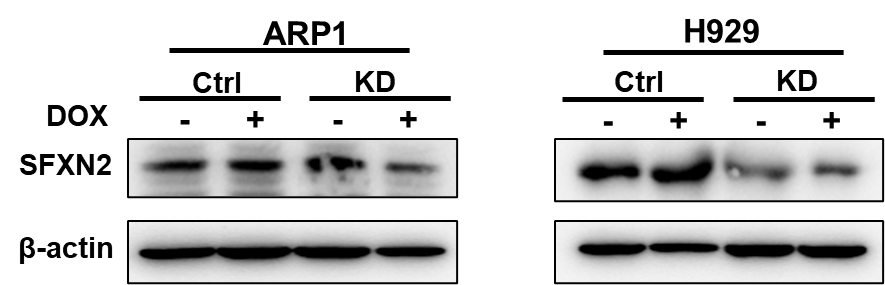


**Fig. S1a Validation of SFXN2 knockdown (KD) in ARP1 and H929 cells relative to control (Ctrl) cells.**


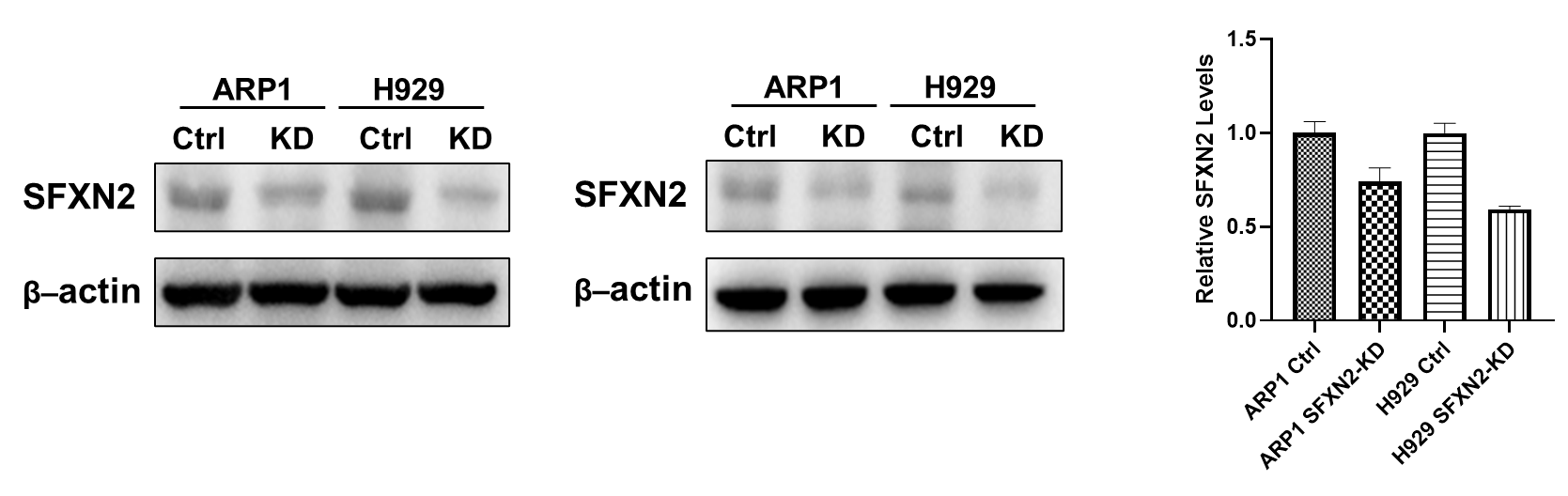


**Fig. S1b SFXN2 expressions in SFXN2-KD ARP1 and H929 cells are determined by WB with gray-scale quantification analysis.**

**Fig. S2 Quantification analysis of WB results indicates increased expression of autophagy related proteins ATG7, ATG5 and LC3, and mitophagy related proteins PINK1 and Parkin post SFXN2 knockdown. Data are shown as mean ± SD for ≥ 3 independent experiments. The statistical significance is set at * *p* < 0.05, ***p* < 0.01, and *** *p* < 0.001 using GraphPad Prism 5 software (GraphPad Software Inc., USA).**

**Fig. S3a Quantification analysis of WB results indicates that the expressions of autophagy related proteins ATG7, Beclin1, ATG5 and LC3 were lower in SFXN2-OE cells than WT cells with EBSS treatment (nutrition-deprived condition).**

**Fig. S3b Quantification analysis of WB results indicates that the expressions of mitophagy related proteins PINK1 and Parkin were lower in SFXN2-OE cells than WT cells with EBSS treatment (nutrition-deprived condition).**


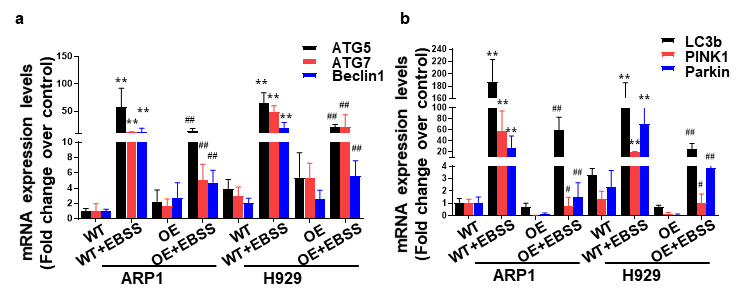


**Fig. S4 SFXN2 suppresses the expressions of starvation-induced autophagy related genes. (a)** RT-qPCR detected the mRNA levels of autophagy biomarkers in EBSS treated ARP1 or H929 cells. **(b)** RT-qPCR detected the mRNA levels of mitophagy related biomarkers in EBSS treated ARP1 or H929 cells.


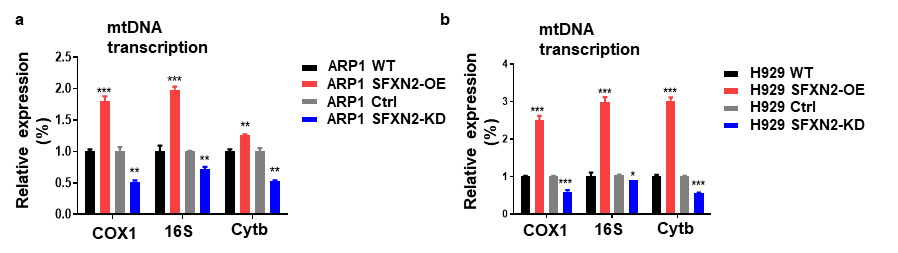


**Fig. S5 RT-qPCR assays test the expressions of mitochondrial encoded genes COX1, cytb and 16S rRNA essential for ATP generation in ARP1 and H929 cells post SFXN2 overexpression or knockdown, respectively.** The expressions of COX1, cytb and 16S rRNA in ARP1 **(a)** and H929 **(b)** WT, SFXN2-OE, SFXN2-Ctrl and SFXN2-KD cells.


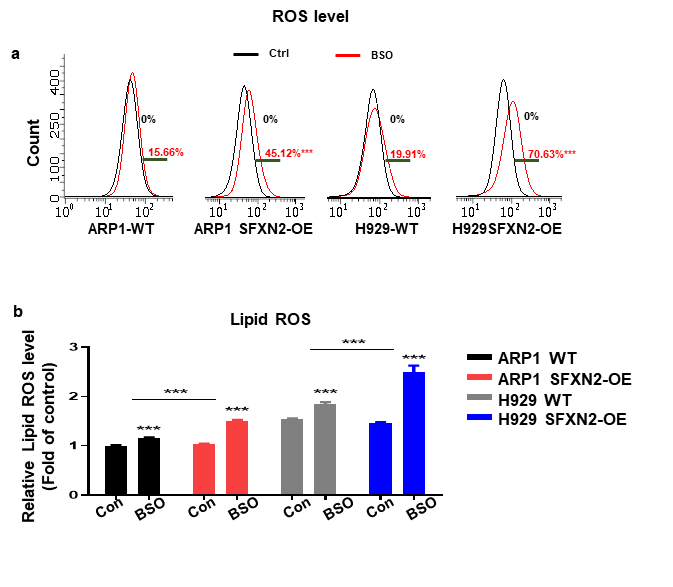


**Fig. S6 Lipid-ROS level detection in WT and SFXN2-OE MM cells upon the treatment of GSH synthesis inhibitor, buthionine sulfoximine (BSO). (a)** Representative ﬂow cytometry results of intracellular ROS levels. **(b)** Quantification of intracellular ROS levels in WT and SFXN2-OE cells treated with BSO.

**
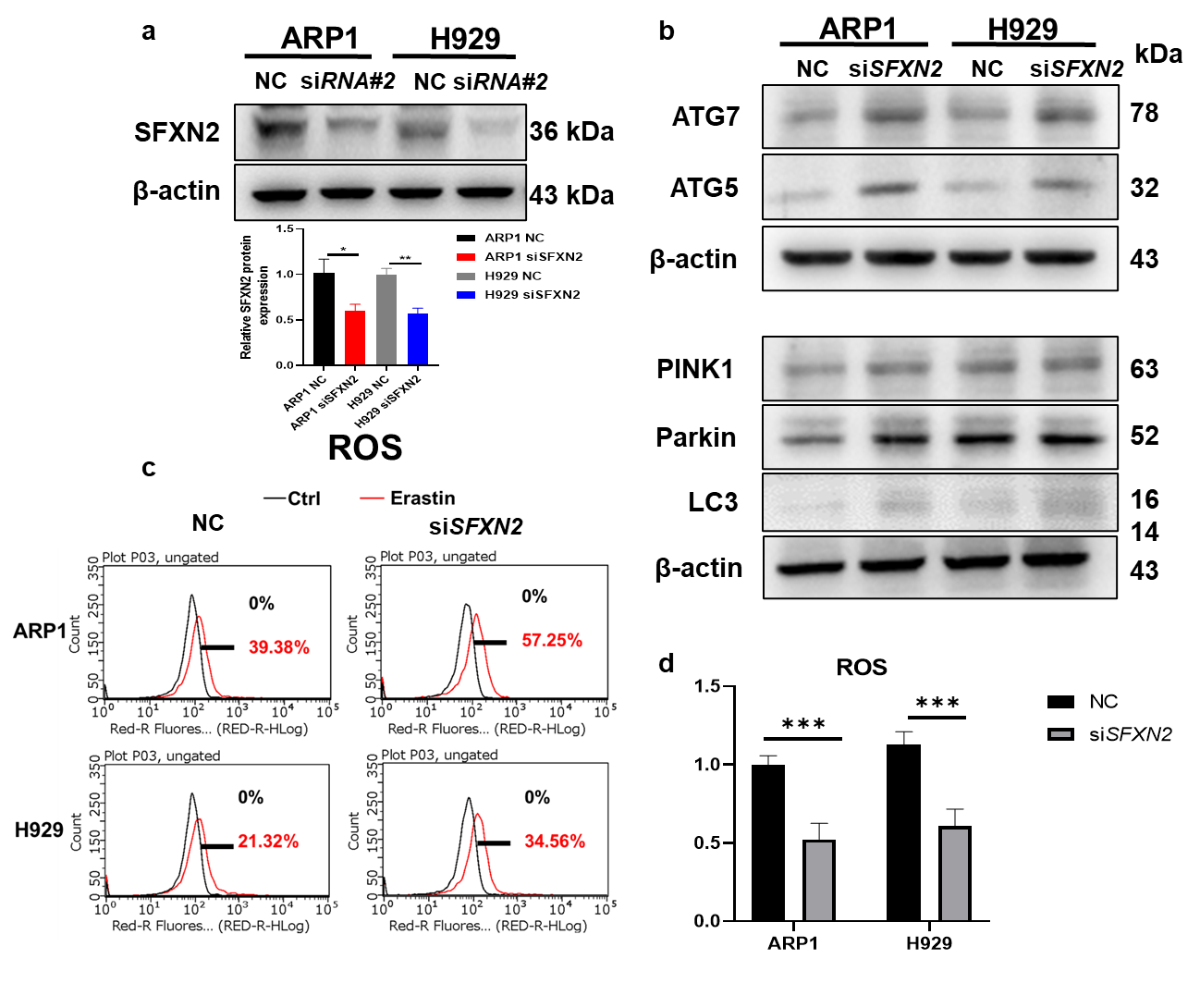
**

**Fig. S7 Knockdown of SFXN2 using siRNA induces elevation of autophagy/mitophagy related proteins and suppresses ROS level.** **(a)** Knockdown efficiency of SFXN2 was validated by WB. **(b)** WB examined the expressions of ATG7, ATG5, PINK1, Parkin and LC3 in NC and siSFXN2 cells. **(c)** Representative ﬂow cytometry results of intracellular ROS levels. **(d)** Quantification analysis of intracellular ROS levels in NC and siSFXN2 cells.


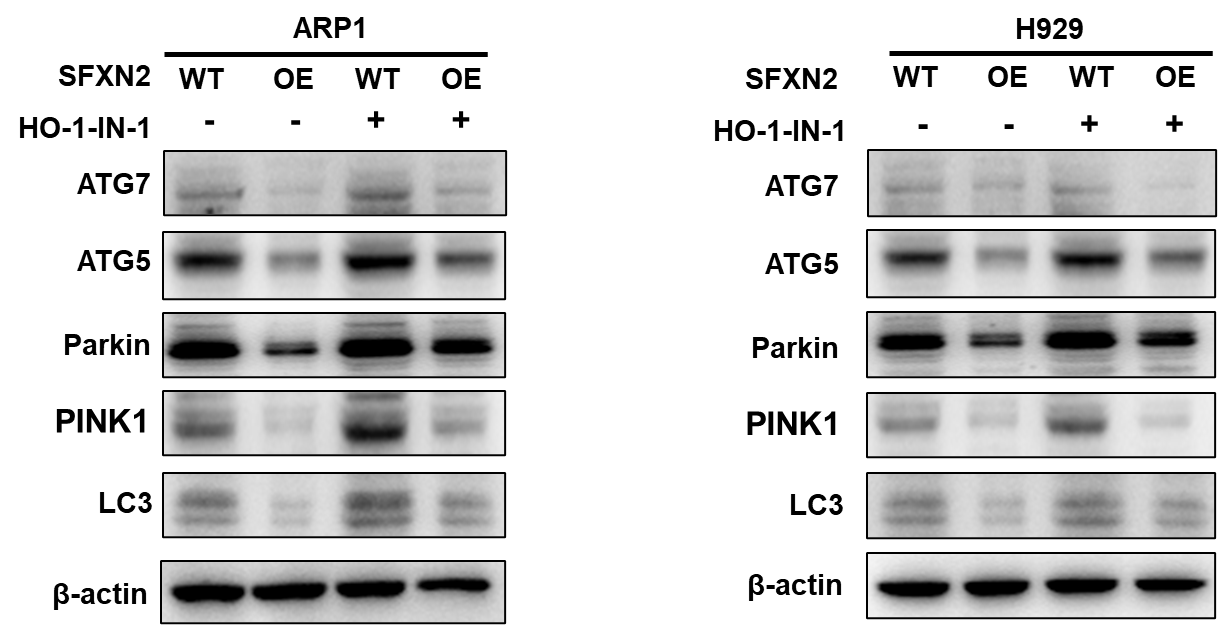


**Fig. S8 The treatment of HO-1-IN1 leads to elevation of autophagy/mitophagy related proteins ATG7, ATG5, LC3, Parkin and PINK1 in SFXN2-OE cells compared to WT cells.**


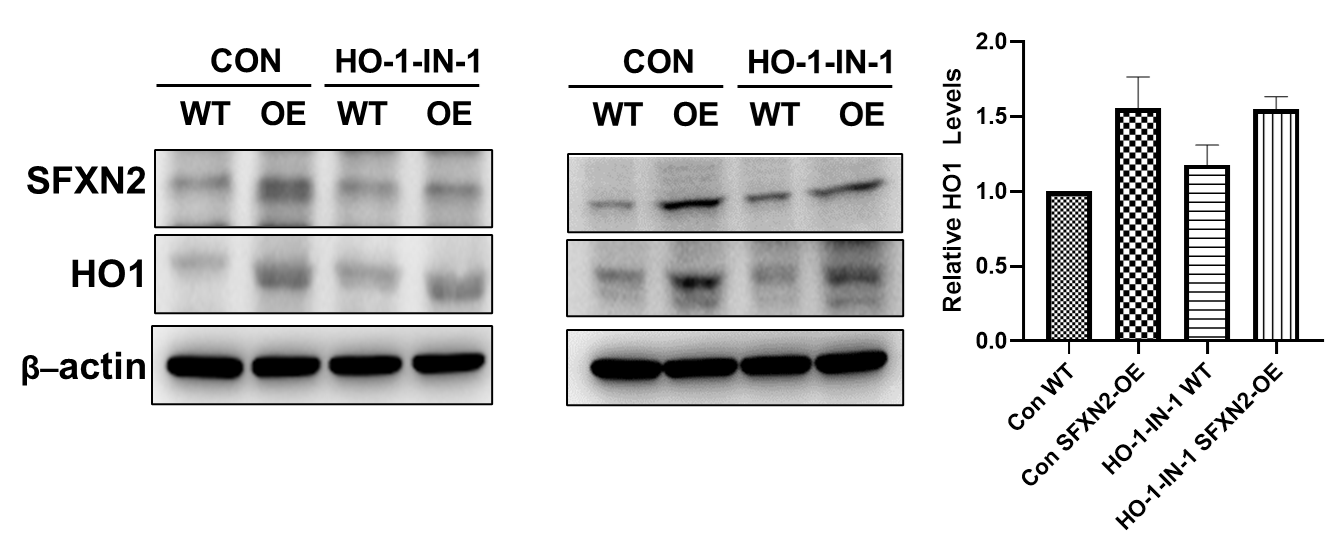


**Fig. S9 Protein levels of HO1 and SFXN2 are determined by WB in SFXN2-OE MM cells treated with or without HO1 inhibitor (HO-1-IN-1).**
