# Supplementary material for: Elevated SFXN2 limits mitochondrial autophagy and increases iron-mediated energy production to promote multiple myeloma cell proliferation
Source: Cell Death Dis. 2022 Sep 26;13(9):822. doi: 10.1038/s41419-022-05272-z (PMC9513108; doi:10.1038/s41419-022-05272-z)
Supplement: Supplementary file 4 — Original Data File [file 41419_2022_5272_MOESM4_ESM.pptx]

## Slide 1
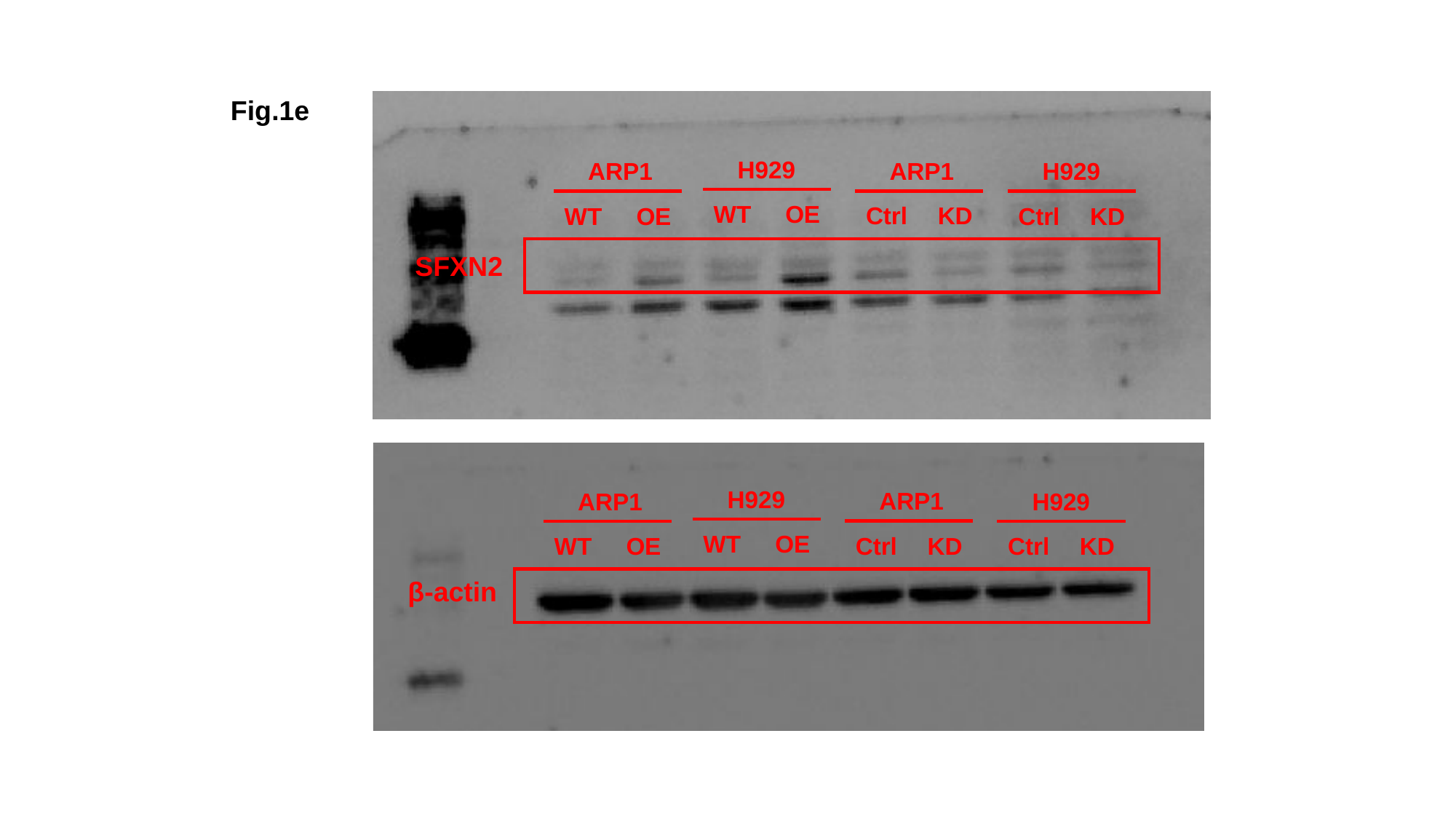

Fig.1e
 H929
WT
OE
 ARP1
Ctrl
KD
 ARP1
WT
OE
 H929
Ctrl
KD
SFXN2
 H929
WT
OE
 ARP1
Ctrl
KD
 ARP1
WT
OE
 H929
Ctrl
KD
β-actin

## Slide 2
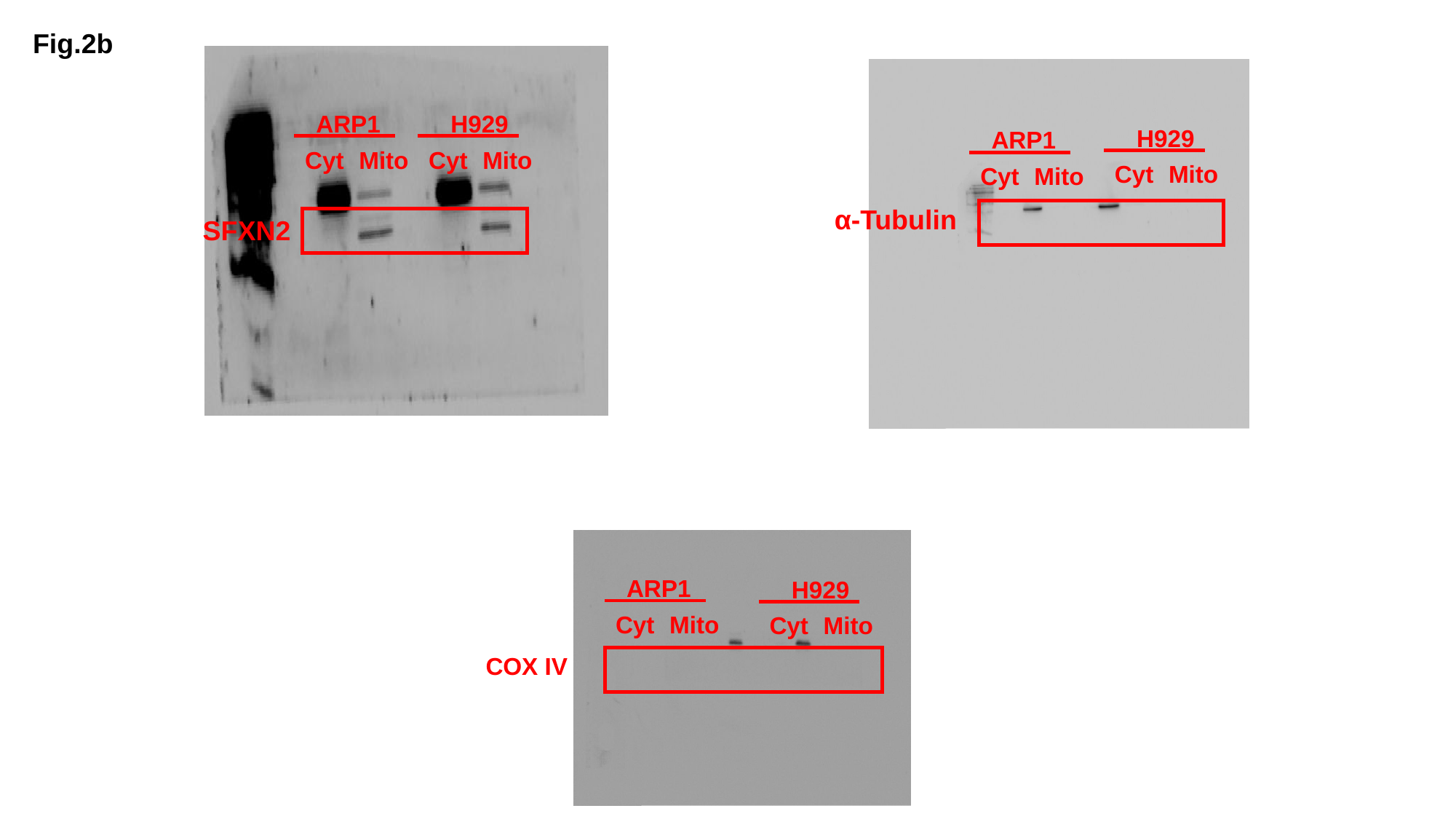

Fig.2b
 H929
Mito
Cyt
 ARP1
Mito
Cyt
SFXN2
 H929
Mito
Cyt
 ARP1
Mito
Cyt
α-Tubulin
 ARP1
Mito
Cyt
 H929
Mito
Cyt
COX IV

## Slide 3
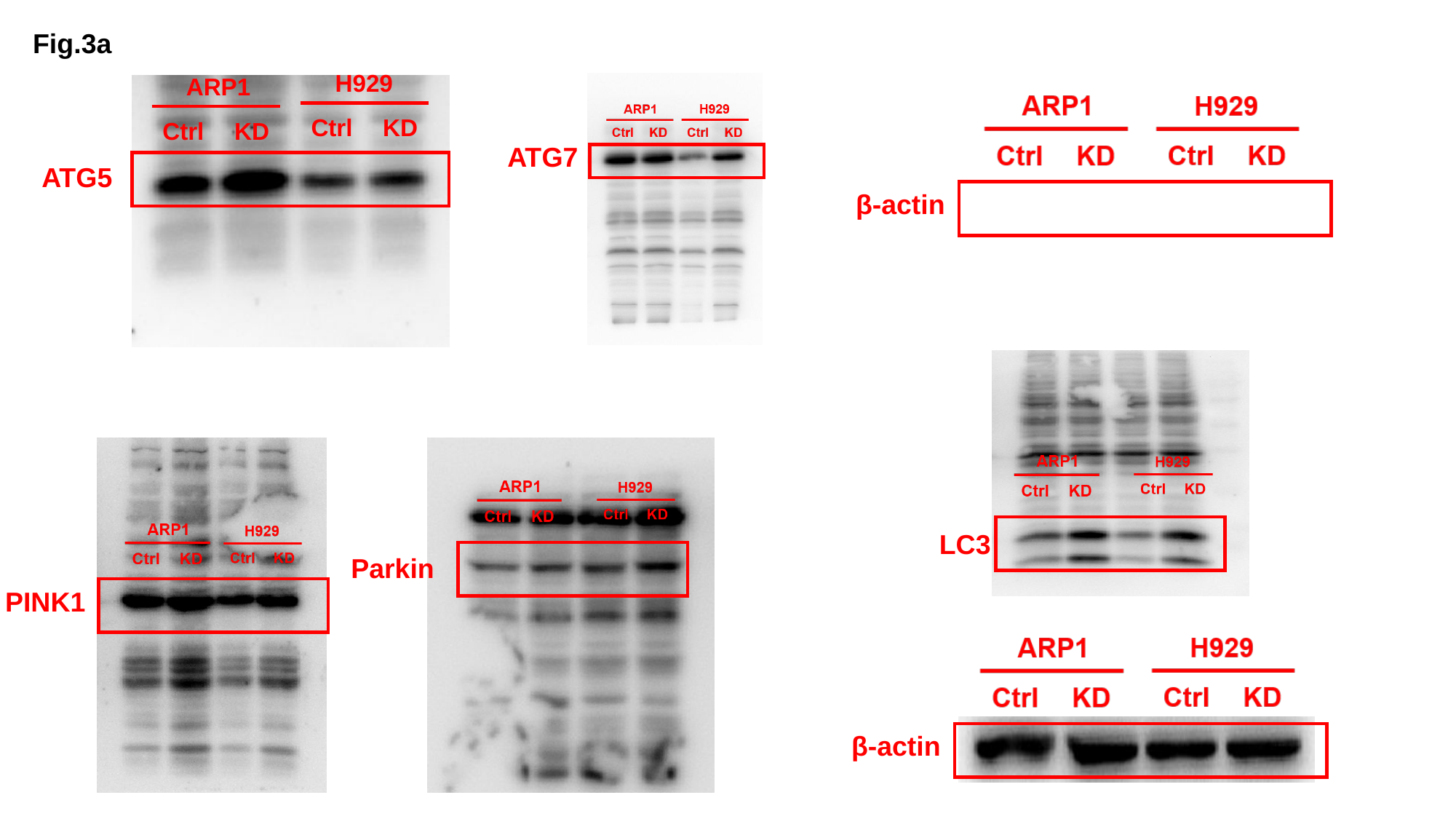

Fig.3a
 H929
Ctrl
KD
 ARP1
Ctrl
KD
β-actin
ATG7
ATG5
LC3
Parkin
PINK1
β-actin

## Slide 4
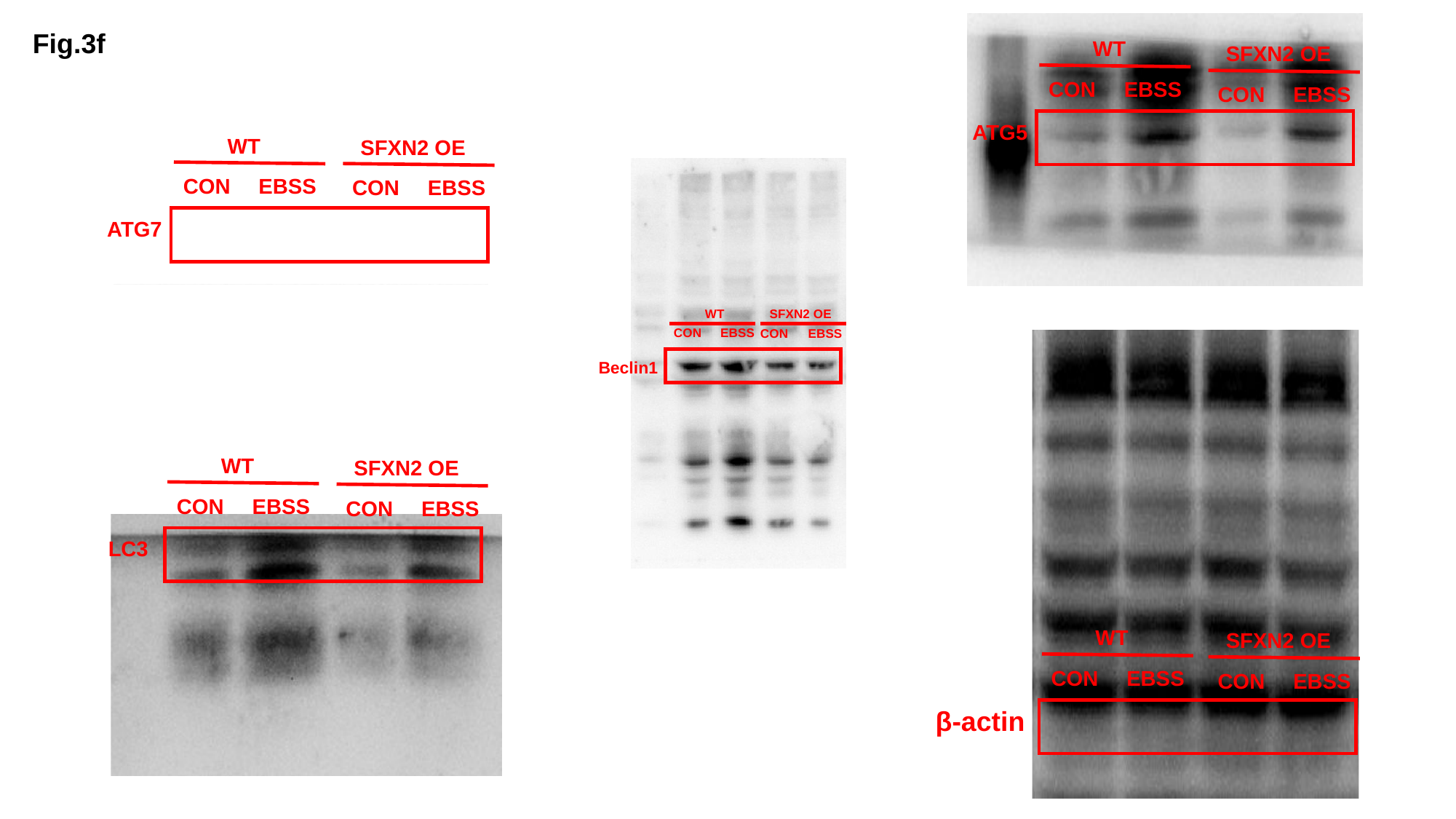

WT
CON
EBSS
SFXN2 OE
CON
EBSS
ATG5
Fig.3f
WT
CON
EBSS
SFXN2 OE
CON
EBSS
ATG7
SFXN2 OE
CON
EBSS
WT
CON
EBSS
Beclin1
WT
CON
EBSS
SFXN2 OE
CON
EBSS
LC3
WT
SFXN2 OE
CON
EBSS
CON
EBSS
β-actin

## Slide 5
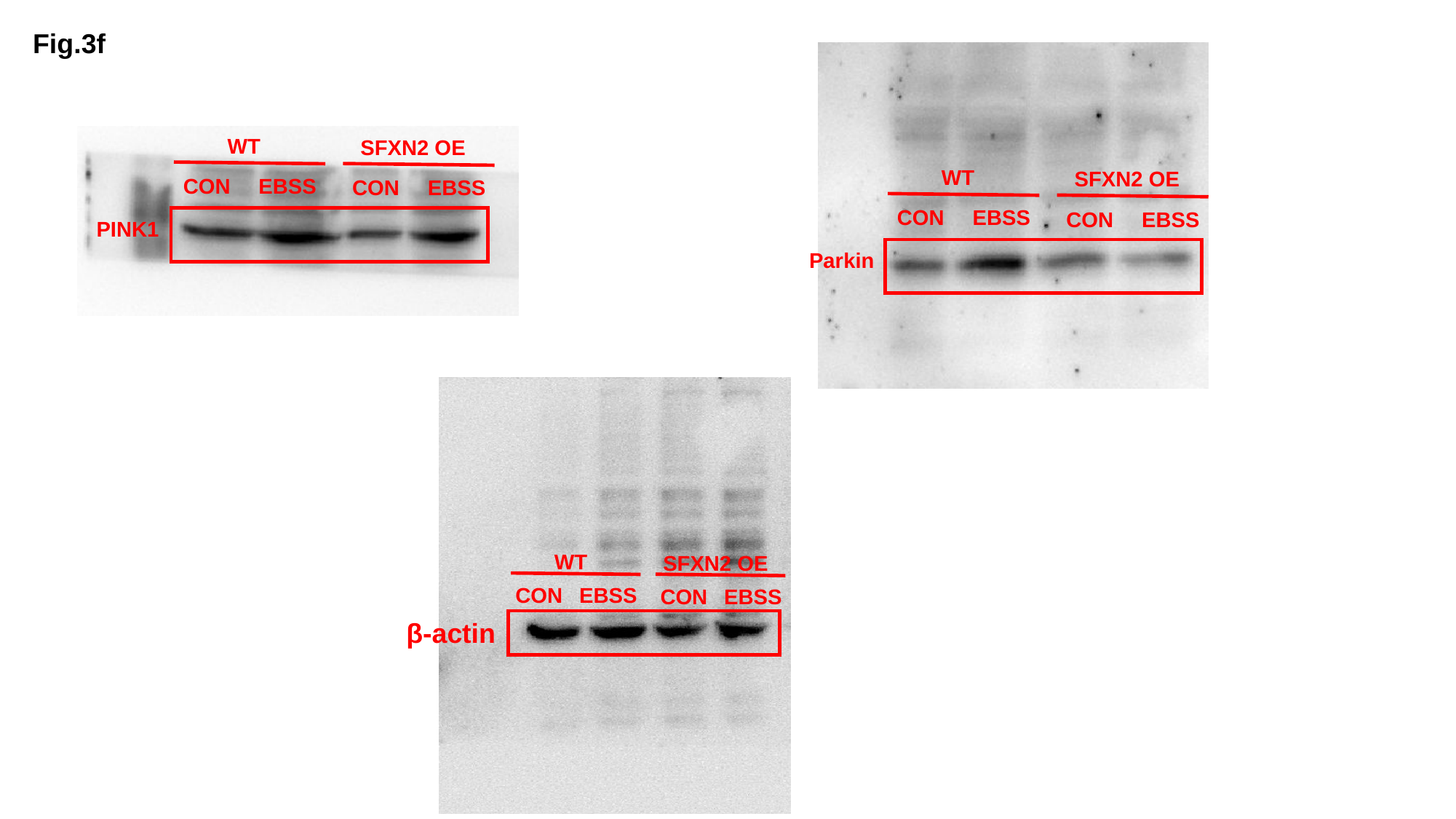

Fig.3f
WT
CON
EBSS
SFXN2 OE
CON
EBSS
Parkin
WT
CON
EBSS
SFXN2 OE
CON
EBSS
PINK1
WT
CON
EBSS
SFXN2 OE
CON
EBSS
β-actin

## Slide 6
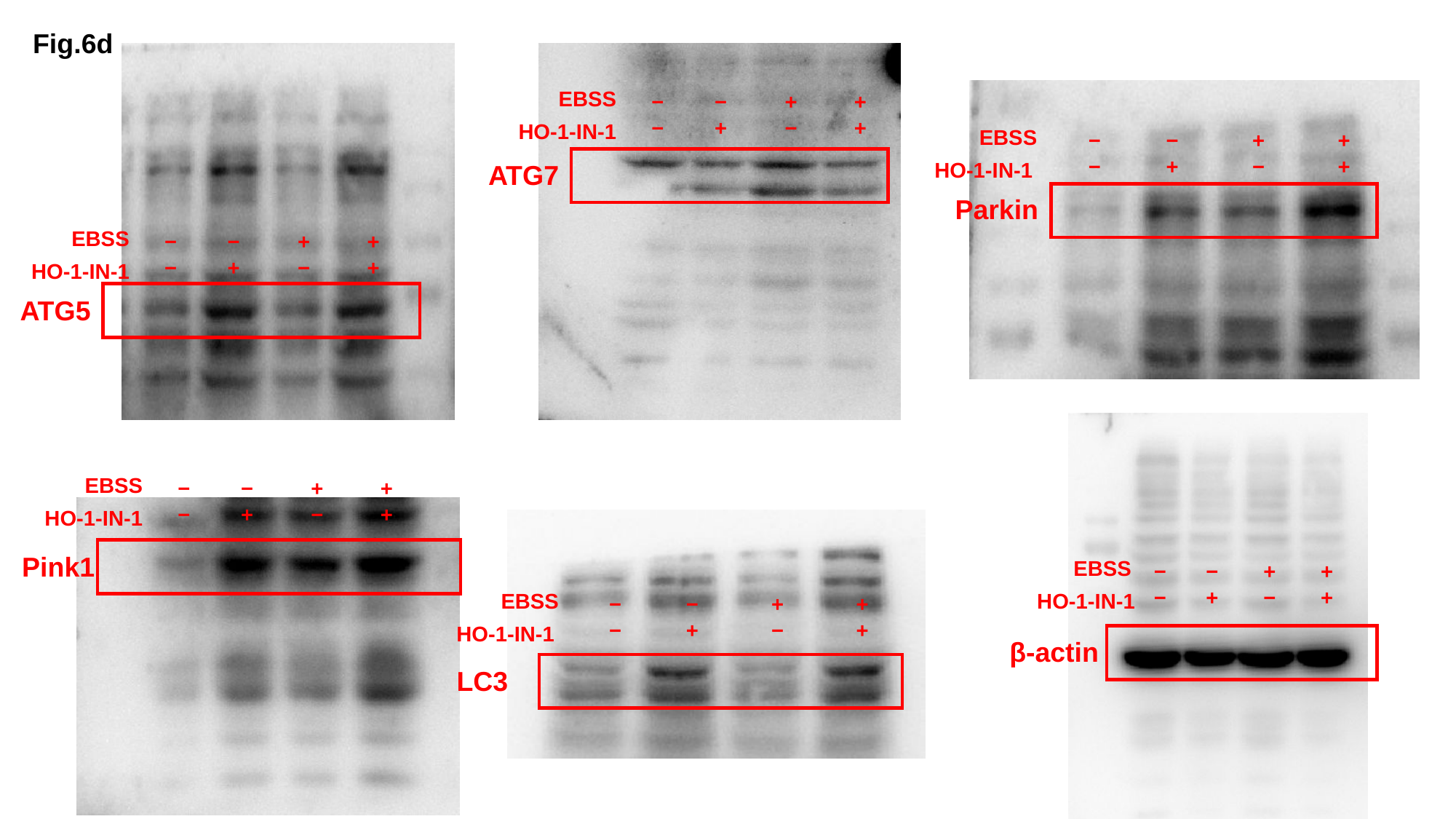

Fig.6d
EBSS
−
−
+
+
−
+
−
+
HO-1-IN-1
ATG7
EBSS
−
−
+
+
−
+
−
+
HO-1-IN-1
Parkin
EBSS
−
−
+
+
−
+
−
+
HO-1-IN-1
ATG5
EBSS
−
−
+
+
−
+
−
+
HO-1-IN-1
Pink1
EBSS
−
−
+
+
−
+
−
+
HO-1-IN-1
EBSS
−
−
+
+
−
+
−
+
HO-1-IN-1
β-actin
LC3

## Slide 7
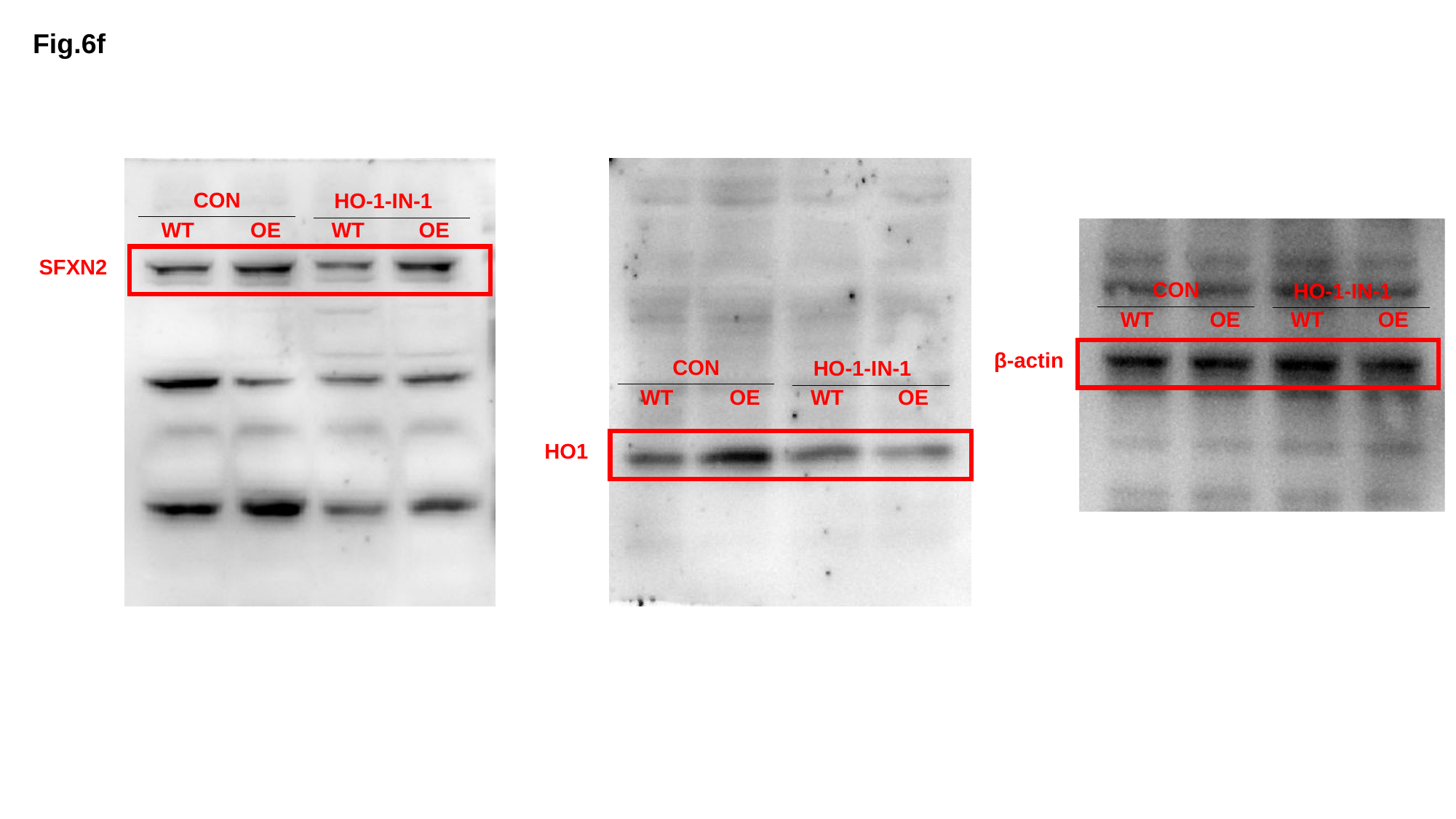

Fig.6f
CON
HO-1-IN-1
WT
OE
WT
OE
CON
HO-1-IN-1
WT
OE
WT
OE
β-actin
SFXN2
CON
HO-1-IN-1
WT
OE
WT
OE
HO1

## Slide 8
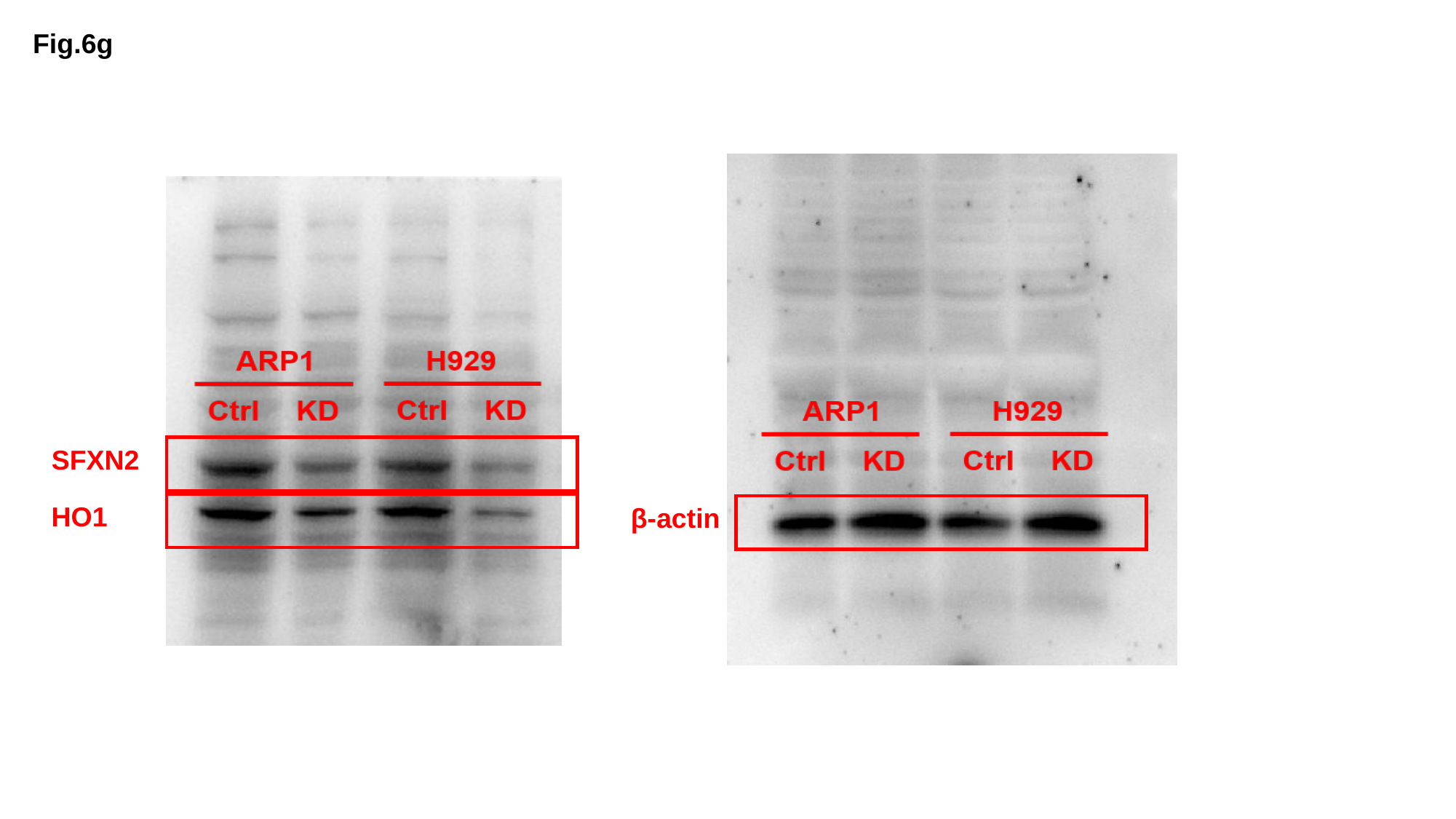

Fig.6g
SFXN2
HO1
β-actin
